# miRNA-1246 in extracellular vesicles secreted from metastatic tumor induces drug resistance in tumor endothelial cells

**DOI:** 10.1038/s41598-021-92879-5

**Published:** 2021-07-05

**Authors:** Chisaho Torii, Nako Maishi, Taisuke Kawamoto, Masahiro Morimoto, Kosuke Akiyama, Yusuke Yoshioka, Takashi Minami, Takuya Tsumita, Mohammad Towfik Alam, Takahiro Ochiya, Yasuhiro Hida, Kyoko Hida

**Affiliations:** 1 Department of Vascular Biology, Hokkaido University Graduate School of Dental Medicine, Sapporo, 060-8586 Japan; 2 Vascular Biology, Frontier Research Unit, Institute for Genetic Medicine, Hokkaido University, Sapporo, 060-0815 Japan; 3 Department of Oral and Maxillofacial Surgery, Hokkaido University Graduate School of Dental Medicine, Sapporo, 060-8586 Japan; 4 Department of Vascular Biology and Molecular Pathology, Hokkaido University Graduate School of Dental Medicine, Sapporo, 060-8586 Japan; 5 Department of Oral Diagnosis and Medicine, Hokkaido University Graduate School of Dental Medicine, Sapporo, 060-8586 Japan; 6 Institute of Medical Science, Tokyo Medical University, Tokyo, 160-0023 Japan; 7 Division of Molecular and Vascular Biology, Institute of Resource Development and Analysis, Kumamoto University, Kumamoto, 860-0811 Japan; 8 Department of Cardiovascular and Thoracic Surgery, Hokkaido University Faculty of Medicine, Sapporo, 060-8638 Japan

**Keywords:** Cancer, Cell biology

## Abstract

Tumor endothelial cells (TECs) reportedly exhibit altered phenotypes. We have demonstrated that TECs acquire drug resistance with the upregulation of P-glycoprotein (P-gp, ABCB1), contrary to traditional assumptions. Furthermore, P-gp expression was higher in TECs of highly metastatic tumors than in those of low metastatic tumors. However, the detailed mechanism of differential P-gp expression in TECs remains unclear. miRNA was identified in highly metastatic tumor extracellular vesicles (EVs) and the roles of miRNA in endothelial cell resistance were analyzed in vitro and in vivo. In the present study, we found that treatment of highly metastatic tumor-conditioned medium induced resistance to 5-fluorouracil (5-FU) with interleukin-6 (IL-6) upregulation in endothelial cells (ECs). Among the soluble factors secreted from highly metastatic tumors, we focused on EVs and determined that miR-1246 was contained at a higher level in highly metastatic tumor EVs than in low metastatic tumor EVs. Furthermore, miR-1246 was transported via the EVs into ECs and induced IL-6 expression. Upregulated IL-6 induced resistance to 5-FU with STAT3 and Akt activation in ECs in an autocrine manner. These results suggested that highly metastatic tumors induce drug resistance in ECs by transporting miR-1246 through EVs.

## Introduction

Tumor blood vessels contribute to tumor growth by providing nutrition and oxygen^1^ and play important roles in tumor progression and metastasis. Anti-angiogenic therapy has been proposed as a novel anti-cancer strategy more than decades ago^2^. Current anti-angiogenic therapies mostly target vascular endothelial growth factor (VEGF) and its receptors, which have prolonged cancer patients’ overall survival. On another note, recent clinical studies have shown the limited results of these drugs. These agents sometimes cause adverse effects, such as lethal hemoptysis^3,4^ and intestinal perforation^5–7^, because the VEGF pathway is also important for normal endothelial cells (NECs)^8^, and drug resistance has been reported. Traditionally, it is considered that the advantage of targeting endothelial cells (ECs) rather than tumor cells is that unlike tumor cells, ECs are genetically stable and do not develop drug resistance^9,10^. However, there have been reports showing that tumor endothelial cells (TECs) themselves may acquire resistance to these drugs^11^. Indeed, cytogenetically abnormal TECs were also detected in malignant tumors, such as lymphoma^12^. We also previously reported that TECs show a degree of cytogenetic abnormalities, such as aneuploidy or abnormal centrosomes, in mouse tumors^13^ and human renal carcinomas^14^. These abnormalities were not caused by tumor cell contamination^13^ but may be affected by the tumor microenvironment. These cytogenetic abnormalities are suggestive of the genetic instability of TECs, predicting the possibility that TECs may acquire drug resistance.

Several TECs are resistant to certain drugs^15,16^. For example, renal carcinoma ECs are resistant to vincristine^15^, whereas hepatocellular carcinoma ECs are resistant to 5-fluorouracil (5-FU)^17^. We recently demonstrated that TECs are resistant to paclitaxel with ABCB1 upregulation, which is a drug transporter. We revealed that VEGF secreted from tumor cells induced P-gp expression in ECs^18,19^. It was also demonstrated that TEC phenotypes, such as drug resistance, chromosomal abnormality, or stemness, are different depending on tumor malignancy^20^, suggesting that tumor-secreting factors may induce resistance in ECs^18^. Naito et al. reported that there are TECs that express P-gp and cause resistance to anti-angiogenic drugs^21^. Taken together, TECs clearly acquire drug resistance; however, the detailed mechanism of acquiring drug resistance in TECs has remained unknown.

Cancer cells have been known to secrete extracellular vesicles (EVs), including exosomes, and these EVs affect tumor progression and metastasis^22–24^. EV is a general term for exosomes or microvesicles that vary from 50 to 1000 nm in diameter. These vesicles contain protein, mRNA, or micro RNA (miRNA) inside, and EVs play a role in intercellular communication^25^. We have reported that tumor-derived EVs enhanced proangiogenic phenotypes in ECs^26^. Regarding the effect of EVs on ECs, tumor-derived EVs reportedly enhanced brain metastasis by altering the brain–blood barrier^27^. Hsu et al. reported that EVs that were secreted from hypoxic lung cancer cells increased angiogenesis and permeability in ECs^28^. However, the mechanisms of TEC abnormality related to tumor EVs still lack elucidation.

Recently, miRNAs have emerged as key regulators of multiple physiologic and pathologic cellular responses, and multiple reports have implicated miRNA in cancer pathogenesis. Additionally, miRNAs represent a class of small (20–25 nucleotides), non-coding RNAs that generally inhibit several target messenger RNAs by repressing the translation of deducing mRNA stability^29^. Furthermore, miRNA is exported to extracellular spaces by EVs or by forming complexes with protein or lipids^30,31^, and then, extracellular miRNAs contribute to cell–cell communication. However, miRNA function in tumor-derived EVs on ECs in primary tumors remains unclear.

In the present study, we identified that miR-1246 was more contained in highly metastatic tumor-derived EVs than in lowly metastatic tumor EVs, and the role of this EV miR-1246 in acquiring drug resistance was elucidated.

## Methods

### Mice

Six week-old female nude mice (BALB/c AJcl-nu/nu, Clea, Japan) were housed under specific-pathogen-free conditions. All procedures for animal care and experimentation adhered to institutional guidelines with the approval of the Hokkaido University Animal Committee and were carried out in compliance with the ARRIVE guidelines.

### Cell lines and culture conditions

Human dermis microvascular endothelial cells (HMVECs) were obtained from Lonza (Lonza, Basel, Switzerland) and cultured in EC growth medium for microvascular cells (EGM-2MV) (Lonza). A375 cells were obtained from American Type Culture Collection (ATCC) (Manassas, VA). A375SM cells (super metastatic human melanoma cells) were kindly provided by Dr. Isaiah J. Fidler, M.D. (Anderson Cancer Center, Houston, TX)^32^. A375 and A375SM cells were cultured in minimum essential medium (MEM) (Gibco, Grand Island, NY) supplemented with 10% heat-inactivated fetal bovine serum (FBS, Sigma, St Louis, MO, USA). Human cervical cancer cell HeLa, human oral squamous carcinoma cell HSC-3, human renal clear cell carcinoma cell OS-RC-2, and human colon carcinoma cell HCT119 were purchased from RIKEN Cell Bank (Tsukuba, Japan). Neonatal human foreskin fibroblast BJ was purchased from ATCC. The Hela, HSC-3, HCT119, and BJ cells were cultured in Dulbecco’s modified eagle medium (Sigma) supplemented with 10% FBS. The OS-RC-2 cells were cultured in RPMI1640 medium (Sigma–Aldrich) with 10% FBS. Normal human epidermal melanocytes were purchased from Kurabo Industries Ltd. (Osaka, Japan) and cultured in melanocyte growth medium (PromoCell, Germany).

The TECs were isolated from tumors that were subcutaneously xenografted with A375 or A375SM. The NECs were isolated from the dermis of tumor-free nude mice, as previously described^13^. Furthermore, ECs were isolated using a magnetic cell sorter device (Miltenyi Biotec, Bergisch Gladbach, Germany) and a flow cytometer (FACS Aria II; BD Biosciences, San Jose, CA, USA) using an anti-CD31 antibody after removing leukocytes with an anti-CD45 antibody. The CD31-positive cells were sorted and seeded on 1.5% gelatin-coated culture plates with EGM-2MV containing 15% FBS. After EC isolation, diphtheria toxin (500 ng/mL; Calbiochem, San Diego, CA, USA) was added to the TEC subcultures to eliminate the remaining human tumor cells and NEC subcultures for technical consistency. The subcultured ECs were sorted through the second round of purification using fluorescein isothiocyanate (FITC)-conjugated *Bandeiraea simplicifolia* lectin–isolectin B4 (Vector Laboratories, Burlingame, CA, USA). The cells were cultured at 37 ℃ in a humidified atmosphere containing 5% CO_2_.

### RT-PCR and real-time PCR

Total RNA was extracted from each EC type using the ReliaPrep RNA Cell Miniprep System (Promega, Madison, WI, USA). First-strand cDNA was synthesized using ReverTra-Plus (Toyobo Co., Osaka, Japan). Real-time RT-PCR was conducted using the KAPA SYBR Fast qPCR Kit (Nippon Genetics, Tokyo, Japan). The cycling conditions were done according to the manufacturer’s instructions, and the CFX Manager (Bio-Rad, Hercules, CA, USA) was used for analysis. The expression levels were normalized to glyceraldehyde-3-phosphate dehydrogenase (GAPDH) levels and were analyzed using the 2^−∆∆**Ct**^. The primers used were as follows: mouse GAPDH: forward, 5′-TCTGACGTGCCGCCTGGAG-3′; reverse, 5′-TCGCAGGAGACAACCTGGTC-3′, human GAPDH: forward, 5′-ACAGTCAGCCGCATCTTCTT-3′; reverse, 5′-GCCCAATACGACCAAATCC-3′, mouse interleukin-6 (IL-6): forward, 5′-AGCTGGAGTCACAGAAGGAGTGGC-3′; reverse, 5′-GGCATAACGCACTAGGTTTGCCGAG-3′, human IL-6: forward, 5′-TACCCCCAGGAGAAGATTCC-3′; reverse, 5′-TTTTCTGCCAGTGCCTCTTT-3′, human androgen receptor (AR): forward, 5′-GACTGCCAGGGACCATGTTTTG-3′; reverse, 5′-GCGCACAGGTACTTCTGTTTCC-3′, human unc-5 netrin receptor B (UNC5B): forward, 5′-ACAAGGCAGAAAGTACCCTCCC-3′; reverse, 5′-GTTCAGGGTCTCCTCATCCAGG-3′, human cathepsin C (CTSC): forward, 5′-AACTGGCCATGAACAGACGTTGGGG-3′; reverse, 5′-AGCTGCCTTGGAGGTAGGTCACCAG-3′.

### Tumor-conditioned medium (CM) preparation

The tumor CM was collected from the culture supernatant of the tumor cells (A375SM or A375 cells) as previously described^18^. Briefly, the tumor cells (1 × 10^6^ cells) were cultured in 10% FBS MEM for 24 h. The tumor CM was obtained from culture supernatant after passing through a 0.22 μm syringe filter (Millipore, Billerica, MA, USA). The control CM was collected from the culture supernatant from HMVECs in 10% FBS MEM in the same way as tumor CM. The HMVECs were treated with tumor CM or control CM in several periods in each experiment. When the cells were treated for 5 days, the medium was changed to freshly collected CM at the third day. The IL-6 level in each CM was measured using an IL-6 ELISA kit (R&D systems, Q6000B, Minneapolis, MN).

### DNA microarray

HMVECs were treated with tumor CM or control CM for 1, 8, and 24 h. Total RNA was extracted from each treated cell as described in the previous text. DNA microarray was conducted according to the manufacturer’s instructions (Thermo Fisher, Affymetrix) as previously described^33^. Data were analyzed according to the MIAME rule. The NCBI accession number for the array data reported in this paper is GSE49426.

### Preparation and characterization of EVs from tumor cell culture supernatant

A375 and A375SM cells (3 × 10^6^ cells) were cultured on a 15 cm-cell culture dish for 48 h in 20 mL of MEM supplemented with 10% EV-depleted FBS. EV-depleted FBS was prepared by ultracentrifugation at 110,000×*g* using Beckman 45Ti rotor for 70 min at 4℃. HMVECs were cultured for 48 h in EBM2 supplemented with 5% EV-depleted FBS. The CM was centrifuged at 2000×*g* for 10 min at 4 ℃, and the supernatant was filtered through a 0.22 μm filter unit (Thermo Scientific Nalgene) to thoroughly remove the cellular debris. Then, to prepare EVs, the supernatant was ultracentrifuged (110,000×*g*, 70 min at 4 ℃) using Beckman 45Ti rotor, the pellet was washed twice with phosphate buffered saline (PBS) by ultracentrifugation at 110,000×*g* using Beckman 45Ti rotor for 70 min at 4 ℃, and EVs were eluted in 200 μL of PBS. The Micro bicinchoninic acid (BCA) Protein Assay Kit (Thermo Scientific, MA, USA) was used to measure the protein concentration. The isolated EVs were characterized through nanoparticle tracking analysis (Nanosight: Quantum Design, Japan) and western blot procedure with anti-CD9 (1:500, Santa Cruz, #59140), anti-CD63 (1:500, Biolegend, #353013), anti-Cytochrome C (1:500, BD Pharmingen, #556433) antibodies, and anti-Mouse Anti-Hsp70 (1:1000, BD Pharmingen, #610607).

We have submitted all relevant data of our experiments to the EV-TRACK knowledgebase (EV-TRACK ID: EV210167)^34^.

### Nanoparticle tracking analysis (NTA)

All NTA measurements were performed using the NS300 unit (Malvern) equipped with a 488 nm laser and the camera sCOMOS. All samples were diluted to provide a concentration of 1 × 10^9^ particles/mL counted using NTA. For each run about 1 mL of the sample was injected into the chamber with a sterile 1-mL syringe controlled via an external syringe pump (syringe pump speed/AU:30). NTA 3.4 software was used for all analysis, using standard settings. In the analysis the mode size (main peak), mean size and its standard derivation values were obtained. All counts were performed in replicates of 5 for each sample, collecting 30 s videos.

### Observation of isolated EVs

The observation of EVs was based on the method reported by Yamashita et al.^35^. EVs were fixed with 4% paraformaldehyde (PFA) for 30 min and then placed on a grid (PVF-C15 STEM Cu150 Grid, Okenshoji, Tokyo, Japan) for 20 min. The grids were treated with 1% glutaraldehyde for 2 min and washed 8 times with dH_2_O. They were stained with 1% uranyl acetate for 10 min. EVs were visualized with a transmission electron microscope (JEM-1400, JEOL, Tokyo, Japan).

### miRNA array

For CM-derived miRNA analysis, the CM collected by culturing normal cells (BJs, melanocytes, and HMVECs) and tumor cells (A375, A375SM, HSC-3, OS-RC-2, and Hela) with serum-free medium (1 × 10^5^ cells, 1 mL/9 cm^2^) for 48 h were used. The CM was centrifuged at 200×*g* for 5 min, and then, the supernatant was further centrifuged at 2000×*g* for 15 min at 4 ℃, and after centrifugation, 200 μL of the supernatant was collected. The total RNA extraction method for miRNA analysis using the miRNeasy mini kit (QIAGEN, Netherlands) is briefly shown in the succeeding text. Then, 200 μL of CM was mixed with 1 mL of QIAZOL (QIAGEN, Netherlands) and allowed to stand for 3 min. The mixed solution was vigorously mixed with 140 μL of chloroform and allowed to stand for 10 min. The mixed solution was centrifuged at 15,000×*g* for 15 min, and 600 μL of the supernatant was collected. Then, 900 μL of ethanol was added to the supernatant, and the miRNA was purified by the same method as mRNA. The final volume of the total RNA solution was adjusted to 30 μL and used for array analysis. EV-derived RNA extraction was carried out in the same method as CM after adjusting the EVs to 50 μg/200 μL with PBS. For comprehensive analysis of CM and EV-derived miRNA, 3-D Gene miRNA Microarray Platform was used (Toray, Japan). The miRNA array data were normalized using the global normalization method and used for subsequent analysis.

### Analysis of miR-1246 level in EVs and cells in vitro

To analyze the miR-1246 level in EVs, the EVs were collected from the CM of NECs (HMVEC) and tumor cells (A375 and A375SM cells) as described in the previous text. QIAZOL was mixed with the EV solution, and cel-miR-39 (Hokkaido System Science, Japan) was added (final concentration: 1 nM). Afterward, the total RNA was extracted as described in the previous text. The cells were seeded on six-well plates at 1 × 10^5^ cells/well to analyze the miR-1246 level. Then, 500 µL of QIAZOL was added to the cells, and the total RNA was extracted as described in the previous text. To analyze the miR-1246 level, real-time RT-PCR was conducted using TaqMan MicroRNA Assays (Applied Biosystems, CA, USA) and a Universal PCR Master Mix II (Applied Biosystems), according to the manufacturer’s instructions. The cycling conditions were done according to the manufacturer’s instructions, and the CFX Manager program (Bio-Rad) was used for analysis. The primers and probes were defined as miR-1246 (Assay ID: CSN1EFS). Cel-miR-39 (Assay ID: 000200; UCACCGGGUGUAAAUCAGCUUG) level was used as the external control and RNU6B (Assay ID; 001093; CGCAAGGATGACACGCAAATTCGTGAAGCGTTCCATATTTTT) was used as internal control.

### Analysis of miR-1246 level in EVs from human blood samples

Human melanoma patients’ serum samples were purchased from BizComJapan, Inc.

400 μL of serum was ultracentrifuged (210,000×*g*, 40 min at 4 ℃) using Beckman SW55Ti rotor, the pellet was washed twice with PBS, and EVs were eluted in 100 μL of PBS. miRNA analysis methods were as described in the previous text, and hsa-miR-451 (Assay ID: 001105; AAACCGUUACCAUUACUGAGUUU) was used as an internal control.

### Uptake of EVs-derived miR-1246

The HMVECs were cultured for 24 h with EV-free EBM2 (serum concentration: 5%) and pretreated with the endocytosis inhibitor dynasore (20 µM) for 2 h. The EVs were added at a concentration of 50 μg/mL, and the miR-1246 level was analyzed through qPCR after 12 h.

### miR-1246 transfection

The HMVECs were seeded on six-well plates at 1 × 10^5^ cells/well. After 24 h, miRIDIAN microRNA Human hsa-miR-1246-Mimic (CN-001040, Dharmacon, GE Dharmacon, Lafayette, CO, USA) and miRIDIAN microRNA Mimic Negative Control (CN-001000-01-05) were transfected into the HMVECs using Lipofectamine RNAiMAX (Invitrogen, Carlsbad, CA, USA) to a final concentration of 50 nM, respectively. After 6 h, the medium was replaced and used for subsequent analysis.

### 3′ UTR reporter assay

A 328-base pair fragment (vector 1) and a 264-base pair fragment (vector 2) from the 3′ UTR of *AR*, which contained the predicted target sequences of miR-1246 (located at positions 4714 to 4719 (vector 1), 5870 to 5874 (vector 2), and 5903 to 5908 (vector 2) of the *AR* 3′ UTR), was cloned through PCR from the total RNA isolated from HEK293 cells. A 3′ polyadenylate overhang was added to the PCR products after 15 min of regular Taq polymerase treatment at 72 ℃. The PCR products were cloned into a pGEM^®^-T Easy Vector system (Promega) and ligated into the Not I site of the 3′ UTR of the *Rennila* luciferase gene in the psiCHECK™-2 plasmid (Promega). The following primer sequences were used (shown 5′ to 3′): For vector 1, forward: CACTGTGTTTGCTAGTGCCC, reverse: CATCAGGTGTGATCTGGAAC. For vector 2, forward: CCCACCTGTCTCTTAGCCTG, reverse: CAAATCTGGCCTGTCACCTC. The HEK293 cells were cultured overnight in 96-well tissue culture plates at densities of 1 × 10^4^ cells/well, respectively, and each construct was co-transfected with hsa-miR-1246 mimic or negative control (NC) mimic using the DharmaFECT Duo Transfection Reagent (Dharmacon, Horizon Discovery). The cells were harvested 48 h after transfection, and the *Rennila* luciferase activity was measured and normalized to the firefly luciferase activity using the Dual-Glo Luciferase Assay System (Promega). Relative luminescence was obtained by normalizing the values against the transfection of the NC mimic group. All assays were repeated at least three times, and the representative results are shown.

### Measurement of dead cells

After 24 h of miR-1246 transfection, the HMVECs were treated with 5-FU (10 μM). The cells were stained with FITC-annexin V and propidium iodide using an Annexin V-FLUOS staining kit (Roche, Basel, Switzerland). The cell death was measured by FACS Aria II (BD) and calculated as relative cell ratio using three different experiments data. The HMVECs were treated with recombinant IL-6 (10 ng/mL) or anti-IL-6 receptor antibody (1 μg/mL) for 5 days and then treated with 5-FU (10 μM).

### Cell survival under 5-FU treatment

Each cell was seeded on 96-well plate at 5 × 10^3^ cells/well in EGM-2MV. After 6 h, 5-FU was added to the medium. Cell survival was measured through MTS [3-(4,5-dimethylthylthiazol-2-yl)-5-(3-carboxymethoxyphenyl)-2-(4-sulfophenyl)-2H-tetrazolium] assay (Promega) at 72 h.

### siRNA transfection

UNC5B or AR siRNA was transfected using lipofectamine transfection reagent (Invitrogen, Tokyo, Japan), according to the manufacturer’s instructions. UNC5B siRNA (UNC5B si#1: 5′-UACGUGUUCACGGGCGAGUCCUAUU-3′, 5′-AAUAGGACUCGCCCGUGAACACGUA-3′, UNC5B si#2: 5′-UCCACAGAGCUCACCUGCAAGAUCU-3′, 5′-AGAUCUUGCAGGUGAGCUCUGUGGA-3′, UNC5B si#3: 5′-GAGGGCCAGAUAUUCCAGCUGCAUA-3′, 5′-UAUGCAGCUGGAAUAUCUGGCCCUC-3′) (HSS137260, 137261, 137262, stealth, Thermo Fisher Scientific, USA) and AR siRNA (AR si#1: 5′-GACUCCUUUGCAGCCUUGCUCUCUA-3′, 5′-UAGAGAGCAAGGCUGCAAAGGAGUC-3′, AR si#2: 5′-GAUGAAGCUUCUGGGUGUCACUAUG-3′, 5′-CAUAGUGACACCCAGAAGCUUCAUC-3′, AR si#3: 5′-CCGGAAGCUGAAGAAACUUGGUAAU-3′, 5′-AUUACCAAGUUUCUUCAGCUUCCGG-3′) (HSS100619, 179972, 179973, stealth, Thermo Fisher Scientific, USA). Non-targeting siRNA (Invitrogen) was used as control.

### Western blotting

The cells and EVs were lysed using radio immunoprecipitation assay (RIPA) buffer (Cell Signaling Technology). The total protein concentration was determined using a BCA Protein Assay kit (Pierce, Rockford, IL, USA). Equal amounts of whole protein extracts (10 μg for EVs and 20 μg for others) were separated on SDS-PAGE gels (10% for STAT3 and Akt and 12% for IL-6 analyses). Western blotting was conducted according to standard methods using antibodies specific for STAT3 (Cell Signaling Technology, 12640S, Beverly, MA), pSTAT3 (Cell Signaling Technology, 9145S), Akt (Cell Signaling Technology, 4685S), pAkt (Cell Signaling Technology, 4060S), IL-6 (Abcam, ab6672), β-actin (Cell Signaling Technology, 4970L), and a horseradish peroxidase (HRP)-conjugated secondary antibody as previously described^20^. The signals were detected using ECL Western Blotting Detection Reagent (GE Healthcare, Little Chalfont, UK) and LAS-4000 Mini image analyzer (FUJIFILM, Tokyo, Japan).

### Statistical analysis

All data are expressed as the mean ± standard deviation of triplicate independent experiments. Statistical significance was determined using unpaired Student’s t test between two groups or one-way ANOVA followed by Tukey’s test. p values less than 0.05 were considered statistically significant.

### Ethics approval and consent to participate

The investigation was conducted in accordance with the ethical standards, the Declaration of Helsinki, and national and international guidelines. All methods have been approved by the Institutional Ethics Committee of Hokkaido University (Sapporo, Hokkaido, Japan), and written informed consent was obtained from each patient before surgery. All procedures for animal care and experimentation adhered to institutional guidelines and were approved by the local animal research authorities.

## Results

### IL-6 upregulation by tumor CM induced drug resistance in ECs in an autocrine manner

**Figure 1:**
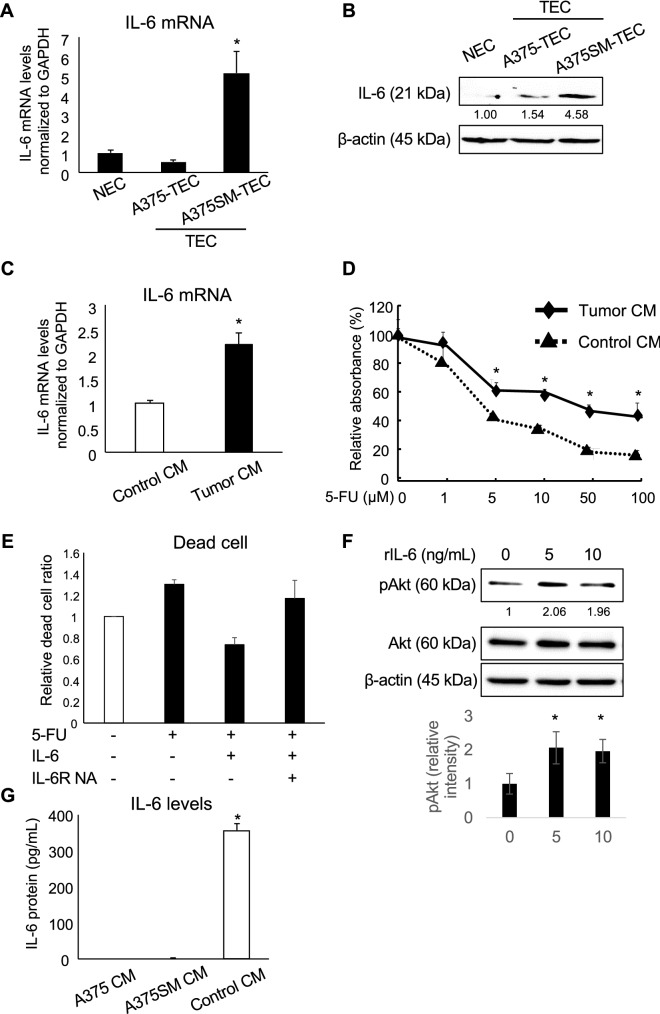
IL-6 upregulation by tumor-conditioned medium induces drug resistance in ECs via Akt activation. (**A**) IL-6 expression was evaluated in mouse normal dermis-derived ECs (NECs), TECs derived from A375 and A375SM tumors (A375-TEC and A375SM-TEC), respectively, through real-time PCR (*P < 0.01 versus NEC and A375-TEC; one-way ANOVA followed by post hoc Tukey’s test; data are presented as mean ± SD, n = 4, real-time RT-PCR runs). (**B**) The levels of IL-6 in mouse normal dermis-derived ECs (NECs), TECs derived from A375 and A375SM tumors (A375-TEC and A375SM-TEC) were determined through western blotting. β-actin was used as an internal control. The value shows the average of relative band intensities, which are taken from densitometric analysis of western blot from three independent experiments. (**C**) IL-6 expression was evaluated in HMVECs treated with conditioned medium (CM) from HMVECs (Control CM) and CM from A375SM (Tumor CM) through real-time PCR after treatment of each CM for 24 h (*P < 0.01 vs. Control CM, two-sided Student’s t-test; data are presented as mean ± SD, n = 4 real-time RT-PCR runs). (**D**) Survival of HMVECs was analyzed through MTS assay under 5-FU treatment condition for 72 h after culture in each CM for 5 days. Control CM and Tumor CM were prepared from HMVECs and A375SM, respectively (*P < 0.01 vs. Control CM, two-sided Student’s t-test; data are presented as mean ± SD, n = 3). (**E**) Dead cells were analyzed through flow cytometry using PI under 5-FU treatment condition for 72 h after treatment with recombinant IL-6 and/or IL-6R neutralizing antibody (NA) in HMVECs for 5 days. Data are presented as mean ± SD, n = 3. (**F**) After stimulation of HMVECs using recombinant IL-6 at the indicated doses, the HMVECs were lysed, and the levels of phosphorylated Akt (pAkt) and total Akt (Akt) were determined through western blotting. The value shows the average of relative band intensities, which are taken from densitometric analysis of Western blot from three independent experiments (*P < 0.05 versus 5 and 10 ng/mL of rIL-6; one-way ANOVA followed by post hoc Tukey’s test). (**G**) IL-6 levels in in each CM were analyzed through ELISA. Control CM was prepared from HMVECs (*P < 0.01 vs. A375CM and control CM, one-way ANOVA followed by post hoc Tukey’s test; data are presented as mean ± SD, n = 3).

We have reported that A375SM-derived tumor CM induced resistance to paclitaxel by upregulating MDR-1/ABCB1 in human NEC:HMVEC^18^. Furthermore, we found that TECs isolated from A375SM tumors (A375SM-TECs) were resistant to 5-FU^17^, which is not a substrate of ABCB1, suggesting the presence of a different mechanism apart from ABCB1. Since IL-6 is a known molecule that is related to drug resistance^36,37^ and was upregulated in A375SM-TECs, we focused on IL-6. The A375SM-TECs showed a significantly higher level of IL-6 than A375 tumor-derived TECs (A375-TECs) or mouse normal dermis-derived ECs (NECs) (Fig. 1A,B). We assumed that A375SM cell-derived factors induced IL-6 upregulation in A375SM-TECs in tumor microenvironment. Then, we treated HMVECs with A375SM-derived CM to check IL-6 expression level. Treatment of tumor CM from A375SM increased IL-6 mRNA expression level in HMVECs (Fig. 1C). The tumor CM from A375SM induced cell survival in HMVECs compared to control, which suggested that the tumor CM from A375SM induced resistance to 5-FU in HMVECs (Fig. 1D). Anti-IL-6 receptor antibody treatment canceled 5-FU resistance induced by IL-6, showing that IL-6 is involved in 5-FU resistance in HMVECs (Fig. 1E). Furthermore, Akt phospholylation level was further increased by 5 ng/mL of IL-6 treatment in HMVEC, although Akt is constitutively activated, because of endothelial cell culture medium containing the several growth factors, such as VEGF (Fig. 1F). It was suggested that IL-6 is also one of additional activator for EC survival. In contrast, IL-6 was not detected in both A375 and A375SM CMs, suggesting that the tumor cells did not secrete IL-6, unlike HMVECs (Control CM) (Fig. 1G). These results suggested that IL-6 was derived from HMVECs after stimulation by tumor secretion factors, but not from tumor cells. Tumor secretion factors induce drug resistance through IL-6 upregulation in HMVECs.

### Tumor EVs induce IL-6 mRNA expression

**Figure 2:**
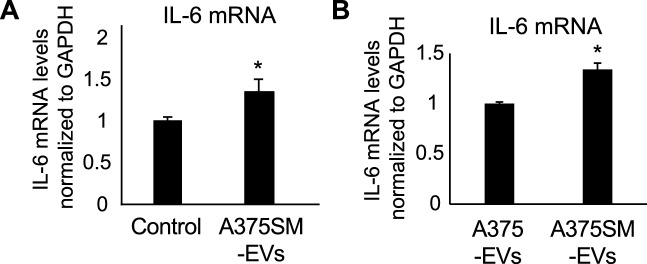
Tumor EVs induces IL-6 mRNA expression. (**A**) IL-6 expression in HMVECs was evaluated through real-time PCR after treatment of HMVECs-derived EVs (Control) or A375SM-derived EVs (A375SM-EVs), respectively, for 24 h (*P < 0.01 vs. Control, two-sided Student’s *t*-test; data are mean ± SD, n = 4 real-time RT-PCR runs). (**B**) IL-6 expression in HMVECs was evaluated through real-time PCR after treatment of each EV for 24 h (*P < 0.01 vs. A375-EVs, two-sided Student’s *t*-test; data are presented as mean ± SD, n = 4 real-time RT-PCR runs).

We focused on EVs as factors that may be contained in tumor CM and induce IL-6 upregulation in ECs. EVs from A375 and A375SM cells were collected and characterized. The size of the isolated EVs was approximately 140 nm in diameter, which was similar to the reported EV size^38^ (Fig. S1A,C). The particle numbers of A375SM EV were more compared to A375 EV (Fig. S1B). Additionally, EV markers, such as HSP70, CD63 and CD9, were detected in the extracted protein from each EV, whereas cytochrome C, a cytoplasm protein, was not detected (Fig. S1D), while tumor cell lysate western blotting showed the expression of all of them. The vesicular structures of the EVs isolated from A375 and A375SM were observed by transmission electron microscopy (Fig. S1E). IL-6 mRNA was upregulated in HMVECs when they were treated with A375SM-EVs, not with HMVECs-derived EVs (Control) (Fig. 2A). Furthermore, the EVs isolated from A375 were added to the HMVEC culture. A375SM-EV treatment induced IL-6 mRNA at a higher level than did A375-EV treatment (Fig. 2B). These data suggested that the factors in A375SM-EVs induced IL-6 mRNA upregulation in HMVECs.

### miR-1246 in EVs from A375SM enhanced IL-6 mRNA expression level in HMVECs

**Figure 3:**
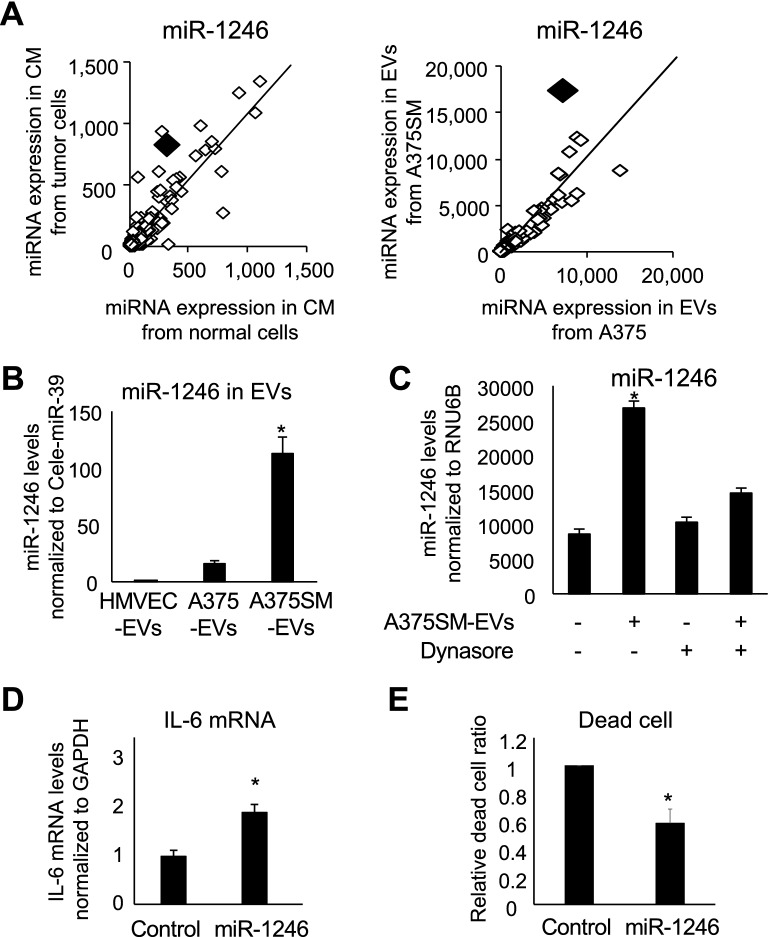
miR-1246 in A375SM EVs enhances IL6 mRNA expression in HMVECs. (**A**) miRNA array analysis in conditioned medium (CM) from tumor and normal cells (left) and in extracellular vesicles (EVs) from A375SM and A375 (right). Each miRNA was plotted. The black rhombus shows miR-1246. miR-1246 was high in both CM from tumor cells and EVs from A375SM. (**B**) miR-1246 expression in each EV was evaluated through real-time PCR (*P < 0.01 vs. HMVEC-EVs and A375-EVs, one-way ANOVA; data are presented as mean ± SD, n = 4 real-time RT-PCR runs). (**C**) miR-1246 expression in HMVECs was evaluated through real-time PCR with or without A375SM-EVs and dynasore (*P < 0.05, one-way ANOVA followed by post hoc Tukey’s test; data are presented as mean ± SD, n = 3 real-time RT-PCR runs). (**D**) IL-6 expression was evaluated through real-time PCR after transfection of miR-1246 in HMVECs. Control miRNA-transfected HMVECs was shown as control (*P < 0.01 vs. control, two-sided Student’s *t*-test; data are presented as mean ± SD, n = 3 real-time RT-PCR runs). (**E**) Dead cells were analyzed through flow cytometry using PI under 5-FU treatment condition for 72 h after transfection of miR-1246 in HMVECs. Control miRNA-transfected HMVECs was shown as Control. Data are presented as mean ± SD, n = 3.

Next, miRNA expression levels were determined to identify the factor in tumor CM or in EVs that induced IL-6 in HMVECs. Here, miRNA was isolated from tumor CM, which was collected from several tumor cell lines (A375, A375SM, HSC3, OS-RC-2, and HeLa) and normal cells (BJs, melanocytes. and HMVECs), and compared with each other through miRNA array. Furthermore, miRNA was also compared between A375SM-EV and A375-EV through miRNA array (Table S1), since we have found that A375SM-TEC showed a higher level of IL-6 than did A375-TEC, as shown in Fig. 1A. Furthermore, miR-1246 was picked up because its level was higher in the CM from tumor cells than in that from normal cells and also in A375SM-EV than in A375-EV (Fig. 3A). Indeed, it was confirmed that miR-1246 was higher in A375SM-EV than in A375-EV or in HMVEC-EV, as determined through PCR (Fig. 3B). The endocytosis inhibitor dynasore canceled miR-1246 induction through A375SM-EV treatment in HMVECs, suggesting that miR-1246 is transported into ECs by A375SM-EVs (Fig. 3C). Furthermore, transfection of miR-1246 in HMVECs increased IL-6 mRNA expression levels (Fig. 3D) consistently with the results of EV treatment, as shown in Fig. 2A. Furthermore, miR-1246 transfection caused resistance to 5-FU in HMVECs (Fig. 3E).

### AR inhibition and induction of drug resistance in HMVECs by miR-1246

**Figure 4:**
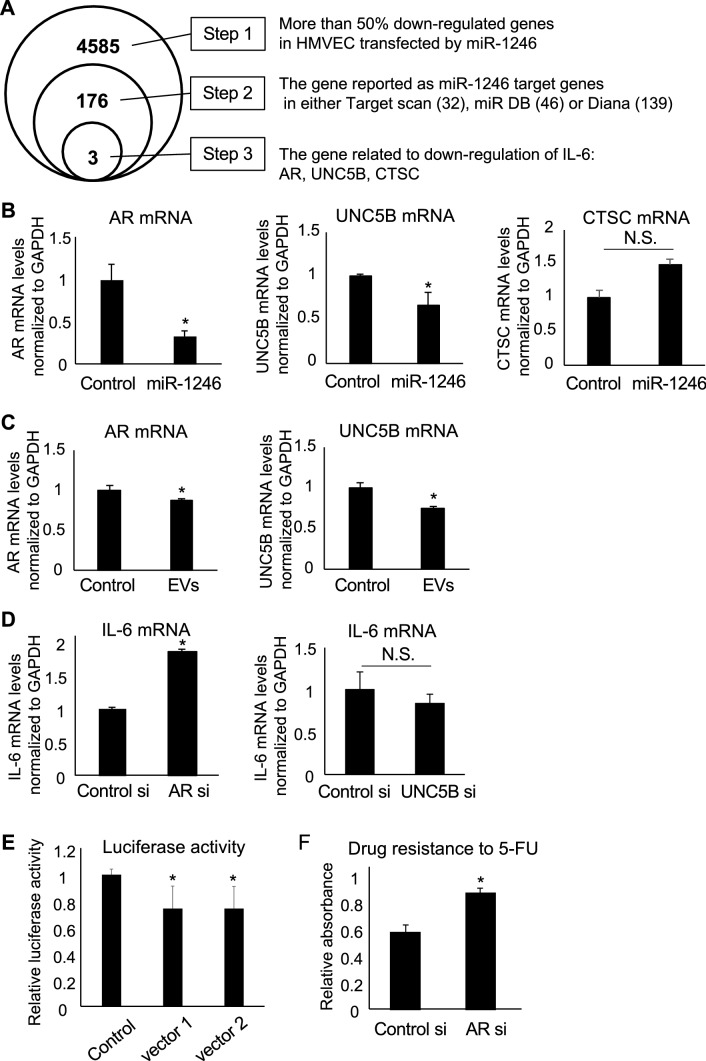
miR-1246 targets androgen receptor (AR) and induces drug resistance in HMVECs. (**A**) Schematic diagram of steps to narrow miR-1246 targets using DNA microarray and IPA. Of note that the three genes AR, UNC5B and CTSC were selected at the Step 3. (**B**) Each mRNA expression was evaluated through real-time PCR after transfection of miR-1246 in HMVECs. Control miRNA-transfected HMVECs was shown as Control (*P < 0.01 vs. control, two-sided Student’s *t*-test; data are presented as mean ± SD, n = 3 real-time RT-PCR runs). (**C**) AR and UNC5B expressions were evaluated through real-time PCR after treatment of A375SM-derived EVs (EVs) cells in HMVECs. HMVEC-EV treated HMVEC was shown as Control (*P < 0.01 vs. control, two-sided Student’s *t*-test; data are presented as mean ± SD, n = 3 real-time RT-PCR runs). (**D**) IL-6 expression was evaluated through real-time PCR after transfection of control, AR or UNC5B siRNA (si) in HMVECs (*P < 0.01 vs. control si, two-sided Student’s *t*-test; data are presented as mean ± SD, n = 3 real-time RT-PCR runs. *N.S.* not significant). (**E**) Luciferase reporter assays with AR 3′ UTR. HEK293 cells were co-transfected with miR-1246 mimic or NC mimic and vector 1, vector 2, or control vector (without AR 3′ UTR). After 48 h, luciferase activities were measured (n = 6 each group). (**F**) Survival of HMVECs was analyzed through MTS assay under 5-FU treatment condition for 72 h after transfection of Control or AR siRNA (*P < 0.01 vs. Control si, two-sided Student’s *t*-test; data are presented as mean ± SD, n = 3).

To address the role of miR-1246 in drug resistance, we attempted to identify the target molecule of miR-1246. First, 4585 genes that were downregulated more than 50% in miR-1246 transfected HMVECs, compared with those in control miR-transfected HMVECs, were selected through DNA microarray analysis (Step 1), as shown in Fig. 4A. Second, among these genes, 176 predicted genes were picked up as miR-1246 targets by three different databases for miRNA target prediction: target scan (32 genes), miR DB (46 genes), and Diana (139 genes) (Step 2). Finally, three genes that were reported to downregulate IL-6 by Ingenuity Pathway Analysis (IPA) were selected as candidate target genes of miR-1246 (Fig. 4A): Androgen receptor (AR), unc-5 netrin receptor B (UNC5B), and Cathepsin C (CTSC). As observed, the mRNA expression levels of AR and UNC5B decreased by miR-1246 transfection in HMVECs; however, CTSC mRNA expression level did not (Fig. 4B). This suggested that AR and UNC5B are targets of miR-1246 but not CTSC. Similarly, A375SM-EVs treatment caused downregulated AR and UNC5B mRNA expressions (Fig. 4C). IL-6 mRNA expression in HMVECs was analyzed after AR or UNC5B inhibition to examine the contribution of these genes on IL-6 expression. IL-6 expression level was increased by the knockdown of AR but not of UNC5B, suggesting that AR suppresses IL-6 expression in HMVECs (Fig. 4D). Furthermore, 3′ UTR reporter assay was conducted to address whether miR-1246 directly binds to the AR gene. The transfection of a vector containing AR 3′UTR decreased luciferase activity. This result showed that miR-1246 binds to the 3′UTR of the AR gene, suggesting that AR is an miR-1246 target (Fig. 4E, Supplementary Fig. S2). Furthermore, we analyzed the effect of AR knockdown on drug resistance to 5-FU in HMVECs by MTS assay. Cell survival of HMVECs under 5-FU treatment increased by AR knockdown, which suggests that AR knockdown caused resistance to 5-FU in HMVECs (Fig. 4F).

### miR-1246 caused drug resistance via activation of STAT3 and Akt

**Figure 5:**
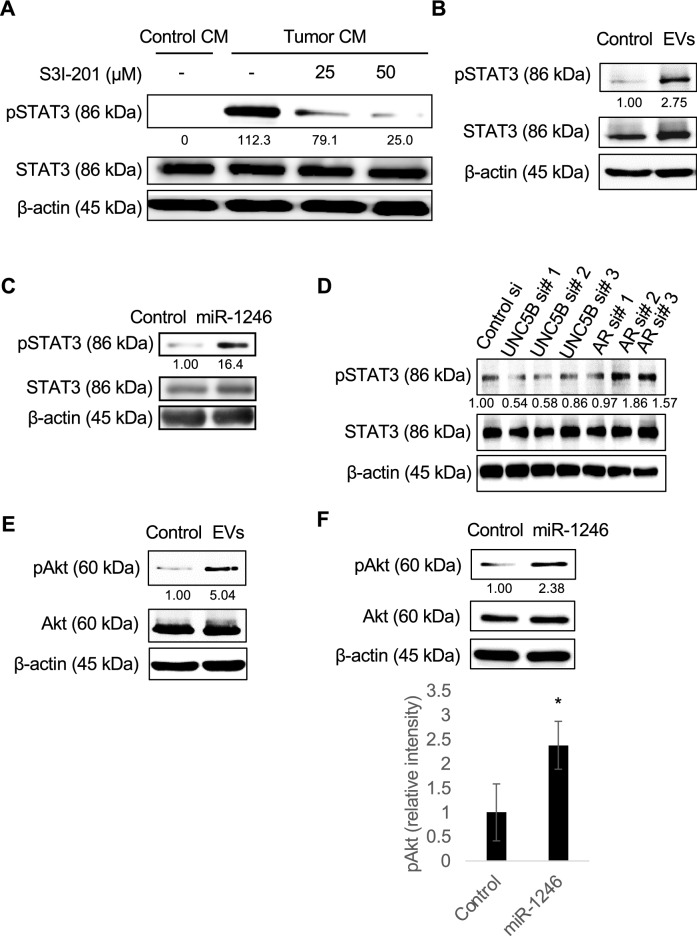
miR-1246 causes drug resistance in HMVECs via STAT3 and Akt activation. (**A**) HMVECs were pre-incubated with S3I-201, a STAT3 phosphorylation inhibitor. After stimulation of control CM from HMVECs or tumor CM from A375SM for 30 min, HMVECs were lysed, and the levels of phosphorylated STAT3 (pSTAT3) and STAT3 were determined through Western blotting. β-actin was used as an internal control. The value shows the average of relative band intensities, which are taken from densitometric analysis of Western blot from three independent experiments. (**B**) After stimulation of A375SM tumor EVs (EVs), the HMVECs were lysed, and the levels of pSTAT3 and STAT3 were determined through Western blotting. PBS was used as Control. β-actin was used as an internal control. The value shows the average of relative band intensities, which are taken from densitometric analysis of Western blot from three independent experiments. (**C**) Control miRNA (Control) or miR-1246 transfected HMVECs were lysed, and the levels of pSTAT3 and STAT3 were determined through western blotting. β-actin was used as an internal control. The value shows the average of relative band intensities, which are taken from densitometric analysis of Western blot from three independent experiments. (**D**) Control, UNC5B or AR siRNA-transfected HMVECs were lysed, and the levels of pSTAT3 and STAT3 were determined through Western blotting. β-actin was used as an internal control. The value shows the average of relative band intensities, which are taken from densitometric analysis of Western blot from three independent experiments. (**E**) After stimulation of A375SM tumor EVs, the HMVECs were lysed and the levels of phosphorylated Akt (pAkt) and Akt were determined through western blotting. β-actin was used as an internal control. The value shows the average of relative band intensities, which are taken from densitometric analysis of Western blot from three independent experiments. (**F**) Control miRNA (Control) or miR-1246-transfected HMVECs were lysed, and the levels of pAkt and Akt were determined through western blotting. β-actin was used as an internal control. The value shows the average of relative band intensities, which are taken from densitometric analysis of Western blot from three independent experiments (*P < 0.05 vs. Control, two-sided Student’s t-test; data are presented as mean ± SD, n = 3).

It is well known that IL-6 activates STAT3, a substance known to be related to drug resistance^39^. Tumor CM induced STAT3 activation, whereas its activation was canceled by S3I-201, a STAT3 inhibitor (Fig. 5A). It was confirmed that tumor EVs (A375SM-EVs) (Fig. 5B) and miR-1246 transfection (Fig. 5C) activated STAT3 in HMVECs. STAT3 phosphorylation levels were not elevated in UNC5B knockdown in HMVECs, but they were increased 1.69 times in AR knockdown (AR si#2) endothelial cells compared to control si (Fig. 5D, Supplementary Fig. S3). Akt activation has been reported to be involved in cell survival or drug resistance^38^. Akt phosphorylation levels was 5 times higher in tumor EV treated in HMVEC compared to control (Fig. 5E). In addition, miR-1246 transfection doubled the phosphorylation level of Akt (Fig. 5F).

### miR-1246 levels in EVs were higher in melanoma patients than in healthy volunteers

**Figure 6:**
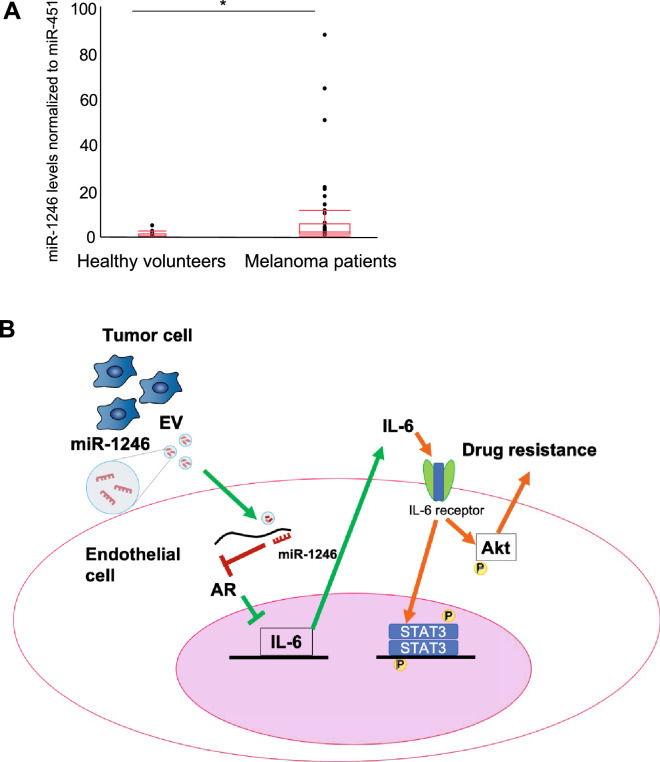
miR-1246 levels in EVs in plasma, and a schematic diagram of the study. (**A**) miR-1246 levels in EVs in healthy volunteers (n = 20) and melanoma patients (n = 42) were determined through real-time PCR (*P < 0.01 vs. healthy volunteers, two-sided Student’s *t*-test; data are presented as mean ± SD, n = 3 real-time RT-PCR runs). (**B**) A schematic diagram of the study. miR-1246 in tumor EVs was taken into endothelial cells, and AR expression was inhibited, which induced IL-6 secretion and caused drug resistance in an autocrine manner through STAT3 and Akt activation.

Finally, EVs were isolated from sera in melanoma patients and healthy volunteers, and miR-1246 levels in EVs were determined. miR-1246 levels were significantly higher in melanoma patients’ EVs than those in healthy volunteers’ EVs (Fig. 6A).

## Discussion

In the present study, we revealed three findings. First, miR-1246 was contained in highly metastatic tumor EVs. Second, miR-1246 in tumor EVs caused IL-6 induction through AR downregulation in ECs. Third, miR-1246 in tumor EVs activated STAT3 and Akt, causing drug resistance in ECs (Fig. 6B).

It has been considered that anti-angiogenic therapy has advantages, because ECs are genetically stable and do not develop drug resistance, unlike tumor cells^10^. However, resistance to this type of therapy has been reported. The major mechanism involved in developing resistance is tumor cell phenotypic change. For example, tumor cells express other angiogenic factors in response to VEGF inhibition^40^. However, we have suggested that another mechanism may be responsible for the abnormalities in TECs. We previously compared the characteristics of TECs with that of NECs and found that TECs contain several abnormalities, such as cytogenetic abnormalities^13,14^ and drug resistance with multidrug resistance 1 (MDR1) upregulation, which is a well-known stem marker and an ABC transporter^18,41^.

Furthermore, we previously demonstrated that TECs are heterogeneous depending on tumor malignancy. Highly metastatic tumor-derived TECs (A375SM-TECs) showed different characteristics from low metastatic tumor-derived TECs (A375-TECs). For example, A375SM-TECs showed higher expression levels of MDR1 than did A375-TECs and showed a more resistant profile to paclitaxel, an anti-cancer drug that is a substrate of ABCB1^20^. We have found that tumor-secreted VEGF-A induced MDR1 mRNA upregulation in NECs through Y box-binding protein 1 activation^18^. These findings suggested that tumor-derived factors can cause EC resistance, and these alterations may be dependent on tumor malignancy. Additionally, TECs showed resistance to 5-FU, which is not a substrate of ABCB1^17^, suggesting that TECs possess other mechanisms of drug resistance besides ABCB1. We focused on IL-6, because IL-6 was upregulated by tumor CM treatment, and it is reportedly involved in drug resistance in tumor cells^36,37,42^. Transcriptional factor C/EBP was activated through the IL-6 autocrine loop mechanism, causing MDR-1 upregulation and inducing resistance to doxorubicin, vincristine, and taxol in IL-6-expressing mammary carcinoma cells^36^. Furthermore, the IL-6 autocrine loop upregulated expressions of anti-apoptotic genes, such as Bcl-2, Bcl-xL, and XIAP, and drug resistance genes, such as MDR-1 and GSTpi, accompanied by the activation of the Ras/MEK/ERK and PI3K/Akt pathways in uterine cancer cells^37^. We previously reported that TECs expressed higher levels of IL-6 than did NECs^43^, and Akt was more activated in TECs than in NECs^43^. In the present study, the IL-6 autocrine loop induced 5-FU resistance by activating STAT3 and Akt in HMVECs. It was suggested that tumor CM-induced resistance in HMVECs was caused by the IL-6 autocrine loop and not by tumor derived-IL-6.

EVs are the generic term for exosomes or microvesicles, which contain biomolecules, such as protein, mRNA, and miRNA, playing roles in intercellular communications^25^. It has been known that tumor cells take advantage of their EV secretion for tumor growth and progression in an autocrine manner^22–24^, and EV secretion was increased by microenvironmental factors, such as hypoxia, low nutrition, and inflammation^44–46^. Furthermore, EVs affect parenchymal cells in a paracrine manner. There are several reports about the effects of tumor EVs on blood vessels. We have reported that tumor-derived EVs were taken into ECs through endocytosis, resulting in pro-angiogenic phenotypes in ECs^26^. A recent study reported that breast cancer cell EVs enhanced brain metastasis by breaking the blood–brain barrier^27^. However, whether tumor EVs affect TECs in the primary site has remained unclear.

It is known that miRNAs are small non-coding RNAs that reduce protein expression through destabilization and/or translational suppression of target RNA (mRNA) molecules^29^. Thus, miRNAs are key regulators of multiple physiologic and pathologic cellular responses. Multiple reports have implicated that miRNA are involved in cancer progression. Furthermore, miRNAs are one of the important biomolecules contained in EVs and exported to extracellular spaces by EVs, playing important roles in intercellular communication^31^. In the present study, we focused on miR-1246 that was contained in highly metastatic tumor-derived EVs. Recent studies related to the function of miR-1246 in cancer cells have been reported^47–52^. For example, miR-1246 inhibits CCNG2 expression, causing drug resistance and stemness in pancreatic cancer cells^50^. Furthermore, miR-1246 in EVs secreted from colon cancer cells were imported into ECs and induced angiogenesis by downregulating promyelocytic leukemia expression, resulting in Smad 1/5/8 activation^47^. However, the function of miR-1246 remains unlabeled. We found that CCNG2 expression was not downregulated by miR-1246 in HMVECs (data not shown), although CCNG2 was reported to be a target of miR-1246 and is involved in drug resistance. It was suggested that miR-1246 have other target molecules in ECs, and AR was found to be one. In cancer biology, there are several reports about AR^53^. It has been reported that AR expression was negative in triple-negative breast carcinoma, and it was suggested that the loss of AR may be correlated with poor prognosis^54^. Additionally, decreased AR expression induced stemness phenotypes in prostate cancer cells through STAT3 activation^55^. Although the role of AR in ECs is still unclear, previous reports may support our findings that AR downregulation induced drug resistance through STAT3 activation and this activation contributed to cell survival^56^. It has been reported that the feedback activation of STAT3 was a side effect of chemotherapy^57^. PI3K/Akt signaling plays an important role in the acquisition of tumors cells’ resistance to anti-cancer drugs^58^.

Although the detailed molecular mechanisms by which highly metastatic tumor-secreted EVs contained higher levels of miR-1246 still remain unclear, our results demonstrated first evidence for tumor-derived EVs causing drug resistance in ECs. Furthermore, in this study we could not completely deny the miRNA1246 in the external contaminants because we did not serum free culture medium to collect tumor EVs. We tried to collect EV after culture with FBS-free medium, but it was difficult to obtain enough amounts of EVs. And we considered that it is important to analyze the communication between actively growing tumor cells and endothelial cells, which resemble tumor microenvironment. That is why we have cultured tumor EVs with 10% FBS (EV-depleted)-containing medium. However, we could detect miR1246 in A375SM-EVs even after RNase treatment, although the level is a half of nontreated EVs. But miR1246 level is 10 times higher in A375SM-EVs than in A375-EVs as described in Fig. 3B, so the biological effect of A375SM-EVs comparing with A375-EVs, which we show in this study, is due to miR1246, at least partially.

Recently, resistance to anti-angiogenic therapy has been reported, and we have demonstrated that ABC transporter upregulation is one of the mechanisms involved in acquiring drug resistance^18,19,41^. However, our results suggest that TECs can acquire resistance to other drugs, such as anti-angiogenic drugs (anti-VEGF drugs) and not just paclitaxel, by increasing IL-6 and Akt or STAT3 activation. Particularly, miR-1246-expressing cancers may produce such results. Our results showed that EVs in the blood of melanoma patients contained more miR-1246 than those of healthy volunteers’ EVs. It would be worth addressing the correlation between EV-miR1246 and prognosis, especially treatment outcomes in the future, although the present study did not investigate the correlation between EV-miR1246 and metastasis or prognosis.

## Conclusion

miR-1246 in tumor EVs caused IL-6 induction through AR downregulation in ECs. In addition, miR-1246 in tumor EVs activated STAT3 and Akt, causing drug resistance in ECs. It was suggested that tumor makes TEC resistant via transporting miR-1246 by EV and keeps blood supply and pathway to metastasize to distant organ.

## Supplementary Information

Supplementary Legends.

Supplementary Figures.

Supplementary Table S1.

## Data Availability

The datasets used and analysed during current study are available from the corresponding author on reasonable request.

## Abbreviations

TECs: Tumor endothelial cells
P-gp: P-glycoprotein
ABCB1: ATP-binding cassette sub-family B member 1
EVs: Extracellular vesicles
5-FU: 5-Fluorouracil
IL-6: Interleukin-6
ECs: Endothelial cells
VEGF: Vascular endothelial growth factor
NECs: Normal endothelial cells
miRNA: Micro RNA
HMVECs: Human dermis microvascular endothelial cells
MEM: Minimum essential medium
FBS: Fetal bovine serum
FITC: Fluorescein isothiocyanate
GAPDH: Glyceraldehyde-3-phosphate dehydrogenase
AR: Androgen receptor
UNC5B: Unc-5 netrin receptor B
CTSC: Cathepsin C
CM: Conditioned medium
PBS: Phosphate buffered saline
BCA: Bicinchoninic acid
NC: Negative control
RIPA: Radio immunoprecipitation assay
HRP: Horseradish peroxidase
IPA: Ingenuity pathway analysis
MDR-1: Multidrug resistance 1
