# Supplementary material for: miRNA-1246 in extracellular vesicles secreted from metastatic tumor induces drug resistance in tumor endothelial cells
Source: Sci Rep. 2021 Jul 5;11:13502. doi: 10.1038/s41598-021-92879-5 (PMC8257582; doi:10.1038/s41598-021-92879-5)
Supplement: Supplementary file 1 — Supplementary Legends. [file 41598_2021_92879_MOESM1_ESM.docx]

**Fig. S1. EV characterization.**

(A) Each EV was analyzed by NanoSight. (B) Particle numbers in each EV were counted by NanoSight (**P* < 0.01 vs. A375-EV, two-sided Student’s *t*-test; data are presented as mean ± SD, n = 4). (C) Particle size in each EV were counted by NanoSight (two-sided Student’s *t*-test; data are presented as mean ± SD, n = 4. N.S.: not significant). (D) EV markers, HSP70, CD63 and CD9, in each EV and in each cell lysates, were determined through western blotting. Cytochrome C was used to show no cell contamination. (E) Representative data of electron microscopy of isolated EVs from A375 or A375SM.

**Fig. S2. miR-1246 may bind to the 3**′**UTR of AR.**

The predicted miR-1246 binding site in the 3′UTR of AR is shown. The bold font shows the seed sequence of miR-1246 (6-mer). The 3′UTR assay was conducted using vectors with the indicated sequences.

**Fig. S3. AR knockdown enhanced STAT3 phosphorylation.**

Control and AR siRNA-transfected HMVECs were lysed, and the levels of pSTAT3 and STAT3 were determined through Western blotting (Fig. 5D). β-actin was used as an internal control. The value shows the average of relative band intensities, which are taken from densitometric analysis of Western blot from three independent experiments (*P < 0.05 vs. control si, two-sided Student’s t-test; data are presented as mean ± SD.
