# Supplementary material for: miRNA-1246 in extracellular vesicles secreted from metastatic tumor induces drug resistance in tumor endothelial cells
Source: Sci Rep. 2021 Jul 5;11:13502. doi: 10.1038/s41598-021-92879-5 (PMC8257582; doi:10.1038/s41598-021-92879-5)

Supplementary Information

miRNA-1246 in extracellular vesicles secreted from metastatic tumor induces drug resistance in tumor endothelial cells

Chisaho Torii, Nako Maishi, Taisuke Kawamoto, Masahiro Morimoto, Kosuke Akiyama, Yusuke Yoshioka, Takashi Minami, Takuya Tsumita, Mohammad Towfik Alam, Takahiro Ochiya, Yasuhiro Hida, Kyoko Hida

Supplementary figure S1

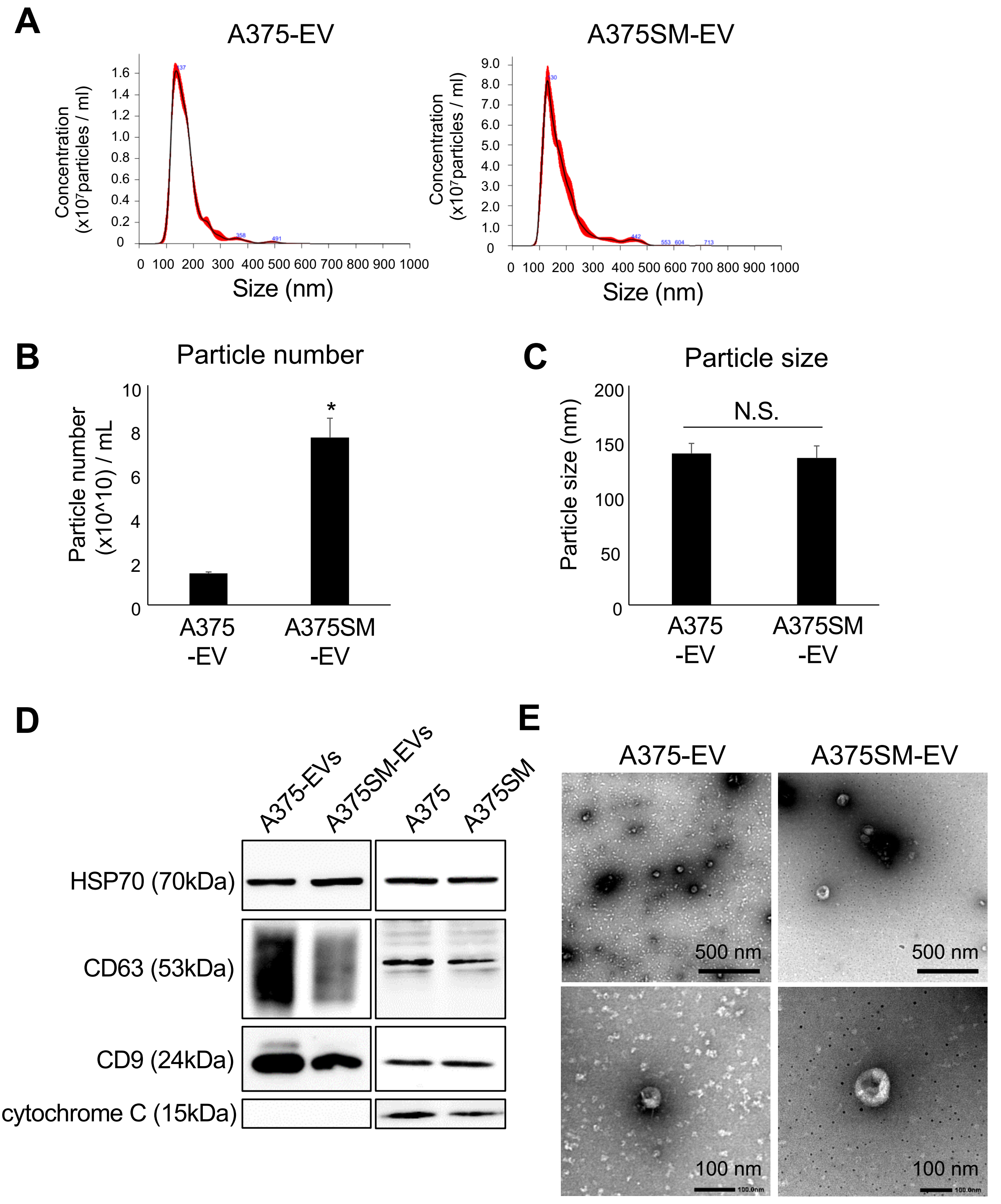

Supplementary figure S2

Vector 1

AR 3'UTR 5'- UACAUUGUAAAUGAAUAUUUGUAUCCAUG -3'

hsa-miR-1246 3'- GGACGAGGUUUU**UAGGUA**A -5'

Vector 2

AR 3'UTR 5'- AAGAUUGUCAUGGAGCUGCAGAUUCCAUU -3'

hsa-miR-1246 3'- ACGAGGUUUUU**AGGUAA** -5'

AR 3'UTR 5'- CCAAAGACUAGAACACACACAUAUCCAUA -3'

hsa-miR-1246 3'- ACGAGGUUUU**UAGGUA**A -5'

Supplementary figure S3

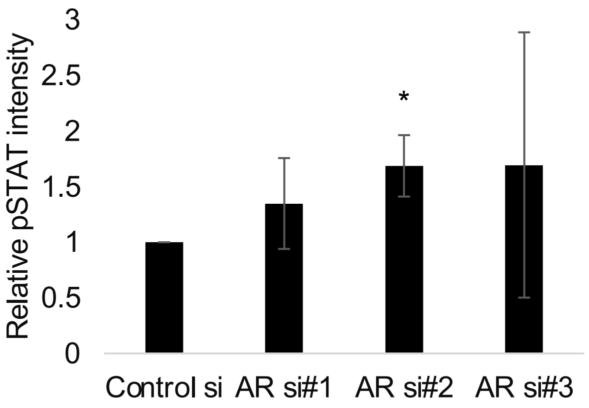

Supplement: Supplementary file 2 — Supplementary Figures. [file 41598_2021_92879_MOESM2_ESM.pdf]
