# Supplementary material for: miRNA-1246 in extracellular vesicles secreted from metastatic tumor induces drug resistance in tumor endothelial cells
Source: Sci Rep. 2021 Jul 5;11:13502. doi: 10.1038/s41598-021-92879-5 (PMC8257582; doi:10.1038/s41598-021-92879-5)
Supplement: Supplementary file 3 — Supplementary Table S1. [file 41598_2021_92879_MOESM3_ESM.docx]

**Table S1**: miRNA expression in EVs from A375 and A375SM.

| Name | ID | A375 EV | A375SM EV | Ratio | Log2Ratio |
| --- | --- | --- | --- | --- | --- |
| hsa-miR-1246 | MIMAT0005898 | 7115.2 | 17314.1 | 2.43 | 1.28 |
| hsa-miR-4459 | MIMAT0018981 | 8915.7 | 12240.9 | 1.37 | 0.46 |
| hsa-miR-3648 | MIMAT0018068 | 9189.7 | 11992.6 | 1.31 | 0.38 |
| hsa-miR-1908 | MIMAT0007881 | 7973.8 | 10692.3 | 1.34 | 0.42 |
| hsa-miR-4454 | MIMAT0018976 | 13788.1 | 8656.9 | 0.63 | -0.67 |
| hsa-miR-4466 | MIMAT0018993 | 6619.2 | 8457.7 | 1.28 | 0.35 |
| hsa-miR-4530 | MIMAT0019069 | 6636.1 | 8442.8 | 1.27 | 0.35 |
| hsa-miR-638 | MIMAT0003308 | 6829.1 | 8189.2 | 1.20 | 0.26 |
| hsa-miR-4688 | MIMAT0019777 | 8852.2 | 6208.8 | 0.70 | -0.51 |
| hsa-miR-4484 | MIMAT0019018 | 6711.1 | 6052.8 | 0.90 | -0.15 |
| hsa-miR-3178 | MIMAT0015055 | 6188.8 | 5555.1 | 0.90 | -0.16 |
| hsa-miR-4787-5p | MIMAT0019956 | 8119.3 | 5426.5 | 0.67 | -0.58 |
| hsa-miR-3665 | MIMAT0018087 | 7127.9 | 5337.5 | 0.75 | -0.42 |
| hsa-miR-2861 | MIMAT0013802 | 4744.0 | 4730.8 | 1.00 | 0.00 |
| hsa-miR-762 | MIMAT0010313 | 5839.9 | 4583.5 | 0.78 | -0.35 |
| hsa-miR-4745-5p | MIMAT0019878 | 3883.0 | 4391.0 | 1.13 | 0.18 |
| hsa-miR-4488 | MIMAT0019022 | 4540.6 | 4328.8 | 0.95 | -0.07 |
| hsa-miR-4725-3p | MIMAT0019844 | 3836.0 | 4327.6 | 1.13 | 0.17 |
| hsa-miR-4516 | MIMAT0019053 | 4481.9 | 3674.5 | 0.82 | -0.29 |
| hsa-miR-3197 | MIMAT0015082 | 5015.1 | 3616.9 | 0.72 | -0.47 |
| hsa-miR-4739 | MIMAT0019868 | 4308.1 | 3379.9 | 0.78 | -0.35 |
| hsa-miR-4508 | MIMAT0019045 | 4620.7 | 2857.4 | 0.62 | -0.69 |
| hsa-miR-4732-5p | MIMAT0019855 | 870.5 | 2425.6 | 2.79 | 1.48 |
| hsa-miR-3656 | MIMAT0018076 | 3061.1 | 2286.0 | 0.75 | -0.42 |
| hsa-miR-4467 | MIMAT0018994 | 2045.1 | 2151.7 | 1.05 | 0.07 |
| hsa-miR-1260b | MIMAT0015041 | 3917.3 | 2087.1 | 0.53 | -0.91 |
| hsa-miR-4281 | MIMAT0016907 | 1613.4 | 2007.2 | 1.24 | 0.32 |
| hsa-miR-3196 | MIMAT0015080 | 3330.5 | 1975.7 | 0.59 | -0.75 |
| hsa-miR-663 | MIMAT0003326 | 2069.3 | 1882.8 | 0.91 | -0.14 |
| hsa-miR-1268 | MIMAT0005922 | 1509.3 | 1859.1 | 1.23 | 0.30 |
| hsa-miR-4463 | MIMAT0018987 | 2016.8 | 1724.7 | 0.86 | -0.23 |
| hsa-miR-1228* | MIMAT0005582 | 1649.0 | 1723.1 | 1.04 | 0.06 |
| hsa-miR-92a-2* | MIMAT0004508 | 1352.3 | 1459.0 | 1.08 | 0.11 |
| hsa-miR-3621 | MIMAT0018002 | 2566.3 | 1450.9 | 0.57 | -0.82 |
| hsa-miR-4734 | MIMAT0019859 | 1686.0 | 1396.9 | 0.83 | -0.27 |
| hsa-miR-1469 | MIMAT0007347 | 1357.1 | 1354.6 | 1.00 | 0.00 |
| hsa-miR-4651 | MIMAT0019715 | 1388.1 | 1303.3 | 0.94 | -0.09 |
| hsa-miR-149* | MIMAT0004609 | 1262.8 | 1200.8 | 0.95 | -0.07 |
| hsa-miR-4749-5p | MIMAT0019885 | 1762.9 | 1196.2 | 0.68 | -0.56 |
| hsa-miR-575 | MIMAT0003240 | 898.9 | 1186.3 | 1.32 | 0.40 |
| hsa-miR-4442 | MIMAT0018960 | 1065.4 | 1174.3 | 1.10 | 0.14 |
| hsa-miR-1280 | MIMAT0005946 | 2286.9 | 1110.2 | 0.49 | -1.04 |
| hsa-miR-720 | MIMAT0005954 | 1634.5 | 1060.5 | 0.65 | -0.62 |
| hsa-miR-1268b | MIMAT0018925 | 690.9 | 889.9 | 1.29 | 0.37 |
| hsa-miR-642b | MIMAT0018444 | 710.2 | 855.5 | 1.20 | 0.27 |
| hsa-miR-92b* | MIMAT0004792 | 745.2 | 852.9 | 1.14 | 0.19 |
| hsa-miR-3940-5p | MIMAT0019229 | 977.3 | 801.5 | 0.82 | -0.29 |
| hsa-miR-4294 | MIMAT0016849 | 1320.2 | 784.3 | 0.59 | -0.75 |
| hsa-miR-3195 | MIMAT0015079 | 549.1 | 774.7 | 1.41 | 0.50 |
| hsa-miR-4505 | MIMAT0019041 | 1020.8 | 731.0 | 0.72 | -0.48 |
| hsa-miR-4665-5p | MIMAT0019739 | 470.2 | 678.3 | 1.44 | 0.53 |
| hsa-miR-3679-5p | MIMAT0018104 | 562.1 | 647.0 | 1.15 | 0.20 |
| hsa-miR-1202 | MIMAT0005865 | 434.2 | 633.8 | 1.46 | 0.55 |
| hsa-miR-1915 | MIMAT0007892 | 663.5 | 589.2 | 0.89 | -0.17 |
| hsa-miR-4258 | MIMAT0016879 | 479.9 | 569.2 | 1.19 | 0.25 |
| hsa-miR-1275 | MIMAT0005929 | 844.4 | 556.3 | 0.66 | -0.60 |
| hsa-miR-4443 | MIMAT0018961 | 1178.5 | 493.9 | 0.42 | -1.25 |
| hsa-miR-3918 | MIMAT0018192 | 350.7 | 477.3 | 1.36 | 0.44 |
| hsa-miR-4741 | MIMAT0019871 | 791.9 | 470.5 | 0.59 | -0.75 |
| hsa-miR-4634 | MIMAT0019691 | 363.4 | 456.1 | 1.26 | 0.33 |
| hsa-miR-4655-5p | MIMAT0019721 | 574.7 | 455.5 | 0.79 | -0.34 |
| hsa-miR-4689 | MIMAT0019778 | 394.1 | 453.2 | 1.15 | 0.20 |
| hsa-miR-711 | MIMAT0012734 | 509.8 | 452.5 | 0.89 | -0.17 |
| hsa-miR-139-3p | MIMAT0004552 | 262.8 | 444.4 | 1.69 | 0.76 |
| hsa-miR-3619-3p | MIMAT0019219 | 227.2 | 430.3 | 1.89 | 0.92 |
| hsa-miR-4723-5p | MIMAT0019838 | 871.8 | 419.2 | 0.48 | -1.06 |
| hsa-miR-4763-3p | MIMAT0019913 | 482.2 | 414.6 | 0.86 | -0.22 |
| hsa-miR-1203 | MIMAT0005866 | 190.9 | 389.5 | 2.04 | 1.03 |
| hsa-miR-1909 | MIMAT0007883 | 311.8 | 355.6 | 1.14 | 0.19 |
| hsa-miR-4286 | MIMAT0016916 | 585.8 | 355.4 | 0.61 | -0.72 |
| hsa-miR-4532 | MIMAT0019071 | 675.9 | 348.7 | 0.52 | -0.95 |
| hsa-miR-1290 | MIMAT0005880 | 173.9 | 346.1 | 1.99 | 0.99 |
| hsa-miR-3937 | MIMAT0018352 | 254.6 | 330.9 | 1.30 | 0.38 |
| hsa-miR-4492 | MIMAT0019027 | 680.2 | 321.1 | 0.47 | -1.08 |
| hsa-miR-4687-3p | MIMAT0019775 | 434.1 | 319.0 | 0.73 | -0.44 |
| hsa-miR-4792 | MIMAT0019964 | 330.9 | 287.1 | 0.87 | -0.21 |
| hsa-miR-3135b | MIMAT0018985 | 502.5 | 283.3 | 0.56 | -0.83 |
| hsa-miR-135a* | MIMAT0004595 | 211.6 | 272.5 | 1.29 | 0.36 |
| hsa-miR-3663-3p | MIMAT0018085 | 346.3 | 268.0 | 0.77 | -0.37 |
| hsa-miR-23a | MIMAT0000078 | 276.8 | 260.6 | 0.94 | -0.09 |
| hsa-miR-887 | MIMAT0004951 | 213.5 | 260.1 | 1.22 | 0.28 |
| hsa-miR-3185 | MIMAT0015065 | 226.1 | 258.4 | 1.14 | 0.19 |
| hsa-miR-423-5p | MIMAT0004748 | 256.4 | 256.5 | 1.00 | 0.00 |
| hsa-miR-1207-5p | MIMAT0005871 | 190.1 | 248.8 | 1.31 | 0.39 |
| hsa-miR-3141 | MIMAT0015010 | 305.4 | 245.0 | 0.80 | -0.32 |
| hsa-miR-4640-5p | MIMAT0019699 | 136.4 | 233.6 | 1.71 | 0.78 |
| hsa-miR-1914* | MIMAT0007890 | 207.4 | 223.6 | 1.08 | 0.11 |
| hsa-miR-23b | MIMAT0000418 | 236.5 | 221.2 | 0.93 | -0.10 |
| hsa-miR-4695-5p | MIMAT0019788 | 244.9 | 219.1 | 0.89 | -0.16 |
| hsa-miR-4327 | MIMAT0016889 | 503.4 | 217.8 | 0.43 | -1.21 |
| hsa-miR-3622a-5p | MIMAT0018003 | 193.8 | 215.1 | 1.11 | 0.15 |
| hsa-miR-4730 | MIMAT0019852 | 217.3 | 214.1 | 0.99 | -0.02 |
| hsa-miR-4665-3p | MIMAT0019740 | 150.2 | 207.6 | 1.38 | 0.47 |
| hsa-miR-3180 | MIMAT0018178 | 243.6 | 201.5 | 0.83 | -0.27 |
| hsa-miR-760 | MIMAT0004957 | 208.7 | 199.2 | 0.95 | -0.07 |
| hsa-miR-4476 | MIMAT0019003 | 196.6 | 194.6 | 0.99 | -0.01 |
| hsa-miR-4674 | MIMAT0019756 | 201.4 | 185.7 | 0.92 | -0.12 |
| hsa-miR-24 | MIMAT0000080 | 188.2 | 179.6 | 0.95 | -0.07 |
| hsa-miR-4758-5p | MIMAT0019903 | 194.0 | 178.0 | 0.92 | -0.12 |
| hsa-miR-4257 | MIMAT0016878 | 367.1 | 176.9 | 0.48 | -1.05 |
| hsa-miR-296-5p | MIMAT0000690 | 169.4 | 170.9 | 1.01 | 0.01 |
| hsa-miR-1225-5p | MIMAT0005572 | 162.5 | 169.3 | 1.04 | 0.06 |
| hsa-miR-1260 | MIMAT0005911 | 323.6 | 166.3 | 0.51 | -0.96 |
| hsa-miR-557 | MIMAT0003221 | 217.1 | 166.2 | 0.77 | -0.38 |
| hsa-miR-4428 | MIMAT0018943 | 116.4 | 159.1 | 1.37 | 0.45 |
| hsa-miR-4417 | MIMAT0018929 | 218.8 | 156.4 | 0.71 | -0.48 |
| hsa-miR-1343 | MIMAT0019776 | 134.4 | 156.0 | 1.16 | 0.21 |
| hsa-miR-4638-5p | MIMAT0019695 | 143.1 | 154.2 | 1.08 | 0.11 |
| hsa-miR-491-5p | MIMAT0002807 | 125.2 | 152.8 | 1.22 | 0.29 |
| hsa-miR-718 | MIMAT0012735 | 129.0 | 152.2 | 1.18 | 0.24 |
| hsa-miR-4667-5p | MIMAT0019743 | 205.3 | 150.0 | 0.73 | -0.45 |
| hsa-miR-4707-3p | MIMAT0019808 | 125.5 | 147.4 | 1.17 | 0.23 |
| hsa-miR-3616-3p | MIMAT0017996 | 233.8 | 146.3 | 0.63 | -0.68 |
| hsa-miR-4763-5p | MIMAT0019912 | 170.2 | 145.1 | 0.85 | -0.23 |
| hsa-miR-187* | MIMAT0004561 | 170.9 | 142.5 | 0.83 | -0.26 |
| hsa-miR-4279 | MIMAT0016909 | 117.9 | 137.1 | 1.16 | 0.22 |
| hsa-miR-744 | MIMAT0004945 | 443.4 | 136.2 | 0.31 | -1.70 |
| hsa-miR-3154 | MIMAT0015028 | 117.9 | 136.2 | 1.15 | 0.21 |
| hsa-miR-4270 | MIMAT0016900 | 146.5 | 135.9 | 0.93 | -0.11 |
| hsa-miR-4740-3p | MIMAT0019870 | 82.2 | 132.2 | 1.61 | 0.69 |
| hsa-miR-21 | MIMAT0000076 | 284.9 | 130.0 | 0.46 | -1.13 |
| hsa-miR-1224-5p | MIMAT0005458 | 114.8 | 128.7 | 1.12 | 0.16 |
| hsa-miR-4728-5p | MIMAT0019849 | 133.5 | 125.0 | 0.94 | -0.10 |
| hsa-miR-4649-5p | MIMAT0019711 | 143.5 | 124.9 | 0.87 | -0.20 |
| hsa-miR-3180-3p | MIMAT0015058 | 178.0 | 121.5 | 0.68 | -0.55 |
| hsa-miR-1976 | MIMAT0009451 | 133.5 | 121.2 | 0.91 | -0.14 |
| hsa-miR-4507 | MIMAT0019044 | 162.5 | 117.3 | 0.72 | -0.47 |
| hsa-miR-4697-5p | MIMAT0019791 | 99.0 | 116.4 | 1.18 | 0.23 |
| hsa-miR-4436b-5p | MIMAT0019940 | 95.4 | 116.2 | 1.22 | 0.28 |
| hsa-miR-146a | MIMAT0000449 | 139.8 | 113.7 | 0.81 | -0.30 |
| hsa-miR-4787-3p | MIMAT0019957 | 134.1 | 112.5 | 0.84 | -0.25 |
| hsa-miR-4687-5p | MIMAT0019774 | 184.1 | 110.7 | 0.60 | -0.73 |
| hsa-miR-4726-5p | MIMAT0019845 | 80.9 | 109.6 | 1.35 | 0.44 |
| hsa-miR-1228 | MIMAT0005583 | 111.4 | 109.1 | 0.98 | -0.03 |
| hsa-miR-4486 | MIMAT0019020 | 116.9 | 108.0 | 0.92 | -0.11 |
| hsa-miR-574-3p | MIMAT0003239 | 96.3 | 108.0 | 1.12 | 0.17 |
| hsa-miR-4534 | MIMAT0019073 | 72.4 | 107.0 | 1.48 | 0.56 |
| hsa-miR-1281 | MIMAT0005939 | 123.7 | 104.8 | 0.85 | -0.24 |
| hsa-miR-3188 | MIMAT0015070 | 110.3 | 103.6 | 0.94 | -0.09 |
| hsa-miR-1229 | MIMAT0005584 | 101.2 | 102.9 | 1.02 | 0.02 |
| hsa-miR-4723-3p | MIMAT0019839 | 80.2 | 102.8 | 1.28 | 0.36 |
| hsa-miR-4695-3p | MIMAT0019789 | 118.5 | 102.2 | 0.86 | -0.21 |
| hsa-miR-4713-5p | MIMAT0019820 | 89.9 | 100.3 | 1.12 | 0.16 |
| hsa-miR-4750 | MIMAT0019887 | 87.8 | 100.2 | 1.14 | 0.19 |
| hsa-miR-92a | MIMAT0000092 | 120.1 | 99.1 | 0.83 | -0.28 |
| hsa-miR-4722-3p | MIMAT0019837 | 84.1 | 97.1 | 1.15 | 0.21 |
| hsa-miR-4449 | MIMAT0018968 | 91.4 | 96.9 | 1.06 | 0.08 |
| hsa-miR-675 | MIMAT0004284 | 129.4 | 96.2 | 0.74 | -0.43 |
| hsa-miR-125b | MIMAT0000423 | 196.0 | 95.1 | 0.49 | -1.04 |
| hsa-miR-1225-3p | MIMAT0005573 | 74.1 | 95.1 | 1.28 | 0.36 |
| hsa-miR-197 | MIMAT0000227 | 89.6 | 92.7 | 1.03 | 0.05 |
| hsa-miR-550a | MIMAT0004800 | 73.8 | 91.8 | 1.24 | 0.31 |
| hsa-miR-4731-5p | MIMAT0019853 | 85.5 | 91.0 | 1.06 | 0.09 |
| hsa-miR-671-3p | MIMAT0004819 | 61.3 | 90.0 | 1.47 | 0.55 |
| hsa-let-7a | MIMAT0000062 | 150.8 | 89.8 | 0.60 | -0.75 |
| hsa-miR-3175 | MIMAT0015052 | 70.6 | 89.2 | 1.26 | 0.34 |
| hsa-miR-4706 | MIMAT0019806 | 79.5 | 88.8 | 1.12 | 0.16 |
| hsa-miR-3682-3p | MIMAT0018110 | 99.2 | 87.9 | 0.89 | -0.17 |
| hsa-miR-103a | MIMAT0000101 | 101.3 | 87.6 | 0.86 | -0.21 |
| hsa-miR-1587 | MIMAT0019077 | 154.3 | 87.0 | 0.56 | -0.83 |
| hsa-miR-615-5p | MIMAT0004804 | 51.2 | 86.4 | 1.69 | 0.76 |
| hsa-miR-1236 | MIMAT0005591 | 81.1 | 86.3 | 1.06 | 0.09 |
| hsa-miR-574-5p | MIMAT0004795 | 85.0 | 85.1 | 1.00 | 0.00 |
| hsa-miR-3928 | MIMAT0018205 | 100.4 | 84.6 | 0.84 | -0.25 |
| hsa-miR-4685-3p | MIMAT0019772 | 116.0 | 84.4 | 0.73 | -0.46 |
| hsa-miR-328 | MIMAT0000752 | 80.5 | 83.9 | 1.04 | 0.06 |
| hsa-miR-513a-5p | MIMAT0002877 | 248.8 | 83.6 | 0.34 | -1.57 |
| hsa-miR-4535 | MIMAT0019075 | 68.4 | 83.2 | 1.22 | 0.28 |
| hsa-miR-370 | MIMAT0000722 | 131.7 | 82.6 | 0.63 | -0.67 |
| hsa-miR-1237 | MIMAT0005592 | 72.5 | 81.4 | 1.12 | 0.17 |
| hsa-miR-765 | MIMAT0003945 | 63.3 | 81.3 | 1.28 | 0.36 |
| hsa-miR-449b* | MIMAT0009203 | 60.9 | 80.6 | 1.32 | 0.40 |
| hsa-miR-3940-3p | MIMAT0018356 | 93.0 | 80.5 | 0.87 | -0.21 |
| hsa-miR-671-5p | MIMAT0003880 | 149.5 | 80.4 | 0.54 | -0.89 |
| hsa-miR-4731-3p | MIMAT0019854 | 63.0 | 80.4 | 1.28 | 0.35 |
| hsa-miR-1913 | MIMAT0007888 | 79.8 | 79.6 | 1.00 | 0.00 |
| hsa-miR-483-3p | MIMAT0002173 | 85.9 | 79.5 | 0.93 | -0.11 |
| hsa-miR-1910 | MIMAT0007884 | 95.0 | 79.4 | 0.84 | -0.26 |
| hsa-miR-100 | MIMAT0000098 | 148.2 | 79.1 | 0.53 | -0.91 |
| hsa-miR-877* | MIMAT0004950 | 74.8 | 78.7 | 1.05 | 0.07 |
| hsa-miR-150* | MIMAT0004610 | 57.3 | 77.8 | 1.36 | 0.44 |
| hsa-miR-1224-3p | MIMAT0005459 | 87.4 | 77.4 | 0.89 | -0.17 |
| hsa-miR-1249 | MIMAT0005901 | 54.4 | 76.7 | 1.41 | 0.50 |
| hsa-miR-92b | MIMAT0003218 | 97.1 | 75.7 | 0.78 | -0.36 |
| hsa-miR-4323 | MIMAT0016875 | 67.0 | 75.5 | 1.13 | 0.17 |
| hsa-miR-4758-3p | MIMAT0019904 | 97.2 | 74.8 | 0.77 | -0.38 |
| hsa-miR-4690-5p | MIMAT0019779 | 104.3 | 71.8 | 0.69 | -0.54 |
| hsa-miR-149 | MIMAT0000450 | 77.3 | 71.5 | 0.93 | -0.11 |
| hsa-miR-16 | MIMAT0000069 | 85.1 | 71.3 | 0.84 | -0.25 |
| hsa-miR-3158-5p | MIMAT0019211 | 47.8 | 71.3 | 1.49 | 0.58 |
| hsa-miR-4646-3p | MIMAT0019708 | 82.9 | 70.8 | 0.85 | -0.23 |
| hsa-miR-4268 | MIMAT0016896 | 51.7 | 70.3 | 1.36 | 0.44 |
| hsa-miR-766 | MIMAT0003888 | 76.0 | 70.2 | 0.92 | -0.11 |
| hsa-let-7d | MIMAT0000065 | 94.7 | 70.0 | 0.74 | -0.44 |
| hsa-miR-4290 | MIMAT0016921 | 90.7 | 70.0 | 0.77 | -0.37 |
| hsa-miR-4447 | MIMAT0018966 | 56.4 | 69.9 | 1.24 | 0.31 |
| hsa-miR-1181 | MIMAT0005826 | 49.7 | 69.6 | 1.40 | 0.49 |
| hsa-miR-2278 | MIMAT0011778 | 57.6 | 69.6 | 1.21 | 0.27 |
| hsa-miR-1296 | MIMAT0005794 | 59.4 | 69.6 | 1.17 | 0.23 |
| hsa-miR-1233 | MIMAT0005588 | 84.8 | 69.3 | 0.82 | -0.29 |
| hsa-miR-4632 | MIMAT0019688 | 57.8 | 69.3 | 1.20 | 0.26 |
| hsa-miR-3651 | MIMAT0018071 | 47.9 | 68.5 | 1.43 | 0.52 |
| hsa-miR-4298 | MIMAT0016852 | 79.5 | 68.0 | 0.86 | -0.22 |
| hsa-miR-3679-3p | MIMAT0018105 | 58.3 | 67.9 | 1.16 | 0.22 |
| hsa-miR-1238 | MIMAT0005593 | 66.1 | 67.5 | 1.02 | 0.03 |
| hsa-miR-668 | MIMAT0003881 | 58.1 | 66.8 | 1.15 | 0.20 |
| hsa-miR-20b | MIMAT0001413 | 70.2 | 66.5 | 0.95 | -0.08 |
| hsa-miR-940 | MIMAT0004983 | 54.2 | 66.3 | 1.22 | 0.29 |
| hsa-miR-3652 | MIMAT0018072 | 64.0 | 66.0 | 1.03 | 0.04 |
| hsa-miR-4728-3p | MIMAT0019850 | 54.4 | 65.1 | 1.20 | 0.26 |
| hsa-miR-4721 | MIMAT0019835 | 59.9 | 64.6 | 1.08 | 0.11 |
| hsa-miR-657 | MIMAT0003335 | 36.9 | 64.3 | 1.74 | 0.80 |
| hsa-miR-920 | MIMAT0004970 | 30.8 | 63.9 | 2.08 | 1.05 |
| hsa-miR-3156-3p | MIMAT0019209 | 48.5 | 63.9 | 1.32 | 0.40 |
| hsa-miR-532-3p | MIMAT0004780 | 60.1 | 63.6 | 1.06 | 0.08 |
| hsa-miR-4430 | MIMAT0018945 | 61.8 | 63.5 | 1.03 | 0.04 |
| hsa-miR-3605-3p | MIMAT0017982 | 55.6 | 63.5 | 1.14 | 0.19 |
| hsa-miR-642a | MIMAT0003312 | 53.3 | 63.3 | 1.19 | 0.25 |
| hsa-miR-3663-5p | MIMAT0018084 | 64.7 | 63.2 | 0.98 | -0.03 |
| hsa-miR-1825 | MIMAT0006765 | 84.3 | 62.9 | 0.75 | -0.42 |
| hsa-miR-3162-5p | MIMAT0015036 | 119.0 | 62.4 | 0.52 | -0.93 |
| hsa-miR-636 | MIMAT0003306 | 45.6 | 61.6 | 1.35 | 0.43 |
| hsa-miR-106a | MIMAT0000103 | 90.7 | 60.7 | 0.67 | -0.58 |
| hsa-miR-4276 | MIMAT0016904 | 68.6 | 60.5 | 0.88 | -0.18 |
| hsa-miR-4515 | MIMAT0019052 | 26.9 | 60.2 | 2.23 | 1.16 |
| hsa-miR-4513 | MIMAT0019050 | 68.7 | 60.1 | 0.87 | -0.19 |
| hsa-miR-17 | MIMAT0000070 | 24.6 | 60.0 | 2.44 | 1.29 |
| hsa-miR-4707-5p | MIMAT0019807 | 74.0 | 59.7 | 0.81 | -0.31 |
| hsa-miR-3917 | MIMAT0018191 | 62.6 | 59.7 | 0.95 | -0.07 |
| hsa-miR-346 | MIMAT0000773 | 50.3 | 59.2 | 1.18 | 0.23 |
| hsa-let-7c | MIMAT0000064 | 110.3 | 57.7 | 0.52 | -0.94 |
| hsa-miR-3622a-3p | MIMAT0018004 | 59.3 | 57.4 | 0.97 | -0.05 |
| hsa-miR-675* | MIMAT0006790 | 51.2 | 56.5 | 1.10 | 0.14 |
| hsa-miR-4271 | MIMAT0016901 | 58.9 | 56.4 | 0.96 | -0.06 |
| hsa-miR-4675 | MIMAT0019757 | 42.5 | 56.2 | 1.32 | 0.40 |
| hsa-miR-30b* | MIMAT0004589 | 32.1 | 56.0 | 1.75 | 0.80 |
| hsa-miR-29a | MIMAT0000086 | 71.0 | 55.8 | 0.79 | -0.35 |
| hsa-miR-764 | MIMAT0010367 | 41.2 | 55.4 | 1.34 | 0.43 |
| hsa-miR-1539 | MIMAT0007401 | 60.5 | 55.4 | 0.92 | -0.13 |
| hsa-miR-615-3p | MIMAT0003283 | 42.9 | 55.2 | 1.29 | 0.36 |
| hsa-miR-637 | MIMAT0003307 | 40.1 | 55.0 | 1.37 | 0.46 |
| hsa-miR-4726-3p | MIMAT0019846 | 62.2 | 54.7 | 0.88 | -0.18 |
| hsa-miR-20a | MIMAT0000075 | 69.2 | 54.6 | 0.79 | -0.34 |
| hsa-miR-1226 | MIMAT0005577 | 37.0 | 54.4 | 1.47 | 0.56 |
| hsa-miR-564 | MIMAT0003228 | 69.8 | 54.3 | 0.78 | -0.36 |
| hsa-miR-4769-3p | MIMAT0019923 | 44.8 | 53.9 | 1.20 | 0.27 |
| hsa-miR-3156-5p | MIMAT0015030 | 15.9 | 53.8 | 3.38 | 1.76 |
| hsa-miR-3131 | MIMAT0014996 | 46.4 | 53.0 | 1.14 | 0.19 |
| hsa-miR-551b | MIMAT0003233 | 16.3 | 52.9 | 3.24 | 1.70 |
| hsa-miR-542-5p | MIMAT0003340 | 66.6 | 52.8 | 0.79 | -0.33 |
| hsa-miR-107 | MIMAT0000104 | 80.3 | 52.7 | 0.66 | -0.61 |
| hsa-miR-663b | MIMAT0005867 | 46.3 | 52.6 | 1.14 | 0.19 |
| hsa-miR-4433 | MIMAT0018949 | 65.0 | 51.8 | 0.80 | -0.33 |
| hsa-miR-193b | MIMAT0002819 | 38.9 | 51.1 | 1.31 | 0.39 |
| hsa-miR-3620 | MIMAT0018001 | 55.1 | 50.9 | 0.92 | -0.11 |
| hsa-miR-3130-5p | MIMAT0014995 | 50.7 | 50.7 | 1.00 | 0.00 |
| hsa-miR-371b-3p | MIMAT0019893 | 44.6 | 50.7 | 1.14 | 0.18 |
| hsa-miR-99a | MIMAT0000097 | 59.2 | 50.7 | 0.86 | -0.22 |
| hsa-miR-1909* | MIMAT0007882 | 54.6 | 50.4 | 0.92 | -0.12 |
| hsa-miR-658 | MIMAT0003336 | 65.5 | 50.2 | 0.77 | -0.38 |
| hsa-miR-3184 | MIMAT0015064 | 66.3 | 49.8 | 0.75 | -0.41 |
| hsa-miR-2277-3p | MIMAT0011777 | 29.8 | 49.5 | 1.66 | 0.73 |
| hsa-miR-3151 | MIMAT0015024 | 32.9 | 49.4 | 1.50 | 0.58 |
| hsa-miR-371-5p | MIMAT0004687 | 39.8 | 49.3 | 1.24 | 0.31 |
| hsa-miR-30d | MIMAT0000245 | 70.9 | 49.1 | 0.69 | -0.53 |
| hsa-miR-486-3p | MIMAT0004762 | 68.6 | 48.7 | 0.71 | -0.50 |
| hsa-miR-4793-3p | MIMAT0019966 | 25.1 | 48.6 | 1.94 | 0.95 |
| hsa-miR-4498 | MIMAT0019033 | 53.1 | 48.5 | 0.91 | -0.13 |
| hsa-miR-4429 | MIMAT0018944 | 70.2 | 48.0 | 0.68 | -0.55 |
| hsa-miR-885-5p | MIMAT0004947 | 30.0 | 48.0 | 1.60 | 0.68 |
| hsa-miR-1234 | MIMAT0005589 | 39.1 | 48.0 | 1.23 | 0.29 |
| hsa-miR-2682* | MIMAT0013518 | 49.7 | 47.3 | 0.95 | -0.07 |
| hsa-miR-3173-5p | MIMAT0019214 | 54.6 | 47.3 | 0.87 | -0.21 |
| hsa-miR-3615 | MIMAT0017994 | 23.1 | 46.9 | 2.03 | 1.02 |
| hsa-miR-4322 | MIMAT0016873 | 37.9 | 46.3 | 1.22 | 0.29 |
| hsa-miR-4716-5p | MIMAT0019826 | 48.4 | 46.3 | 0.96 | -0.06 |
| hsa-miR-614 | MIMAT0003282 | 45.8 | 46.2 | 1.01 | 0.02 |
| hsa-miR-4685-5p | MIMAT0019771 | 28.0 | 45.6 | 1.63 | 0.71 |
| hsa-miR-378f | MIMAT0018932 | 42.9 | 45.0 | 1.05 | 0.07 |
| hsa-miR-181a | MIMAT0000256 | 49.5 | 44.8 | 0.90 | -0.15 |
| hsa-miR-3944-3p | MIMAT0018360 | 45.1 | 44.5 | 0.99 | -0.02 |
| hsa-miR-4700-3p | MIMAT0019797 | 51.3 | 44.4 | 0.87 | -0.21 |
| hsa-miR-4656 | MIMAT0019723 | 98.8 | 44.4 | 0.45 | -1.16 |
| hsa-miR-625* | MIMAT0004808 | 24.2 | 44.2 | 1.83 | 0.87 |
| hsa-miR-4265 | MIMAT0016891 | 35.4 | 43.9 | 1.24 | 0.31 |
| hsa-miR-4440 | MIMAT0018958 | 47.5 | 43.7 | 0.92 | -0.12 |
| hsa-miR-323-5p | MIMAT0004696 | 18.0 | 43.5 | 2.42 | 1.28 |
| hsa-miR-19b | MIMAT0000074 | 53.2 | 43.3 | 0.81 | -0.30 |
| hsa-miR-583 | MIMAT0003248 | 36.4 | 43.0 | 1.18 | 0.24 |
| hsa-miR-4667-3p | MIMAT0019744 | 44.7 | 42.1 | 0.94 | -0.09 |
| hsa-miR-494 | MIMAT0002816 | 119.3 | 41.7 | 0.35 | -1.52 |
| hsa-miR-326 | MIMAT0000756 | 45.0 | 41.5 | 0.92 | -0.12 |
| hsa-miR-4708-3p | MIMAT0019810 | 25.2 | 41.1 | 1.63 | 0.70 |
| hsa-miR-3614-5p | MIMAT0017992 | 36.2 | 41.0 | 1.13 | 0.18 |
| hsa-miR-1307 | MIMAT0005951 | 47.9 | 40.3 | 0.84 | -0.25 |
| hsa-miR-320a | MIMAT0000510 | 65.1 | 40.2 | 0.62 | -0.69 |
| hsa-miR-3190 | MIMAT0015073 | 43.1 | 40.1 | 0.93 | -0.10 |
| hsa-miR-486-5p | MIMAT0002177 | 46.7 | 40.0 | 0.86 | -0.22 |
| hsa-miR-22 | MIMAT0000077 | 39.1 | 39.9 | 1.02 | 0.03 |
| hsa-miR-4455 | MIMAT0018977 | 40.7 | 39.9 | 0.98 | -0.03 |
| hsa-miR-4698 | MIMAT0019793 | 38.4 | 39.9 | 1.04 | 0.05 |
| hsa-miR-629* | MIMAT0003298 | 26.3 | 39.7 | 1.51 | 0.59 |
| hsa-miR-1471 | MIMAT0007349 | 48.8 | 39.6 | 0.81 | -0.30 |
| hsa-miR-4642 | MIMAT0019702 | 30.9 | 39.5 | 1.28 | 0.36 |
| hsa-miR-612 | MIMAT0003280 | 41.1 | 39.2 | 0.95 | -0.07 |
| hsa-miR-4304 | MIMAT0016854 | 35.4 | 39.1 | 1.10 | 0.14 |
| hsa-miR-3646 | MIMAT0018065 | 41.1 | 38.7 | 0.94 | -0.09 |
| hsa-miR-2110 | MIMAT0010133 | 36.7 | 38.7 | 1.05 | 0.08 |
| hsa-miR-199a-5p | MIMAT0000231 | 27.0 | 38.6 | 1.43 | 0.52 |
| hsa-miR-222 | MIMAT0000279 | 70.3 | 38.6 | 0.55 | -0.87 |
| hsa-miR-484 | MIMAT0002174 | 33.3 | 38.6 | 1.16 | 0.21 |
| hsa-miR-4297 | MIMAT0016846 | 47.9 | 38.5 | 0.80 | -0.31 |
| hsa-miR-4321 | MIMAT0016874 | 28.7 | 38.5 | 1.34 | 0.42 |
| hsa-miR-4269 | MIMAT0016897 | 33.8 | 38.3 | 1.13 | 0.18 |
| hsa-miR-1538 | MIMAT0007400 | 28.6 | 38.2 | 1.34 | 0.42 |
| hsa-miR-1200 | MIMAT0005863 | 16.3 | 37.9 | 2.33 | 1.22 |
| hsa-miR-106b | MIMAT0000680 | 34.1 | 37.9 | 1.11 | 0.15 |
| hsa-miR-221 | MIMAT0000278 | 84.3 | 37.9 | 0.45 | -1.15 |
| hsa-miR-584 | MIMAT0003249 | 29.7 | 37.8 | 1.28 | 0.35 |
| hsa-miR-4697-3p | MIMAT0019792 | 40.7 | 37.8 | 0.93 | -0.11 |
| hsa-miR-3622b-3p | MIMAT0018006 | 22.7 | 37.7 | 1.66 | 0.73 |
| hsa-miR-483-5p | MIMAT0004761 | 32.4 | 37.5 | 1.16 | 0.21 |
| hsa-miR-4648 | MIMAT0019710 | 43.0 | 37.5 | 0.87 | -0.20 |
| hsa-miR-4539 | MIMAT0019082 | 44.2 | 36.9 | 0.83 | -0.26 |
| hsa-miR-4732-3p | MIMAT0019856 | 47.6 | 36.7 | 0.77 | -0.37 |
| hsa-miR-192 | MIMAT0000222 | 23.5 | 36.5 | 1.55 | 0.64 |
| hsa-miR-2392 | MIMAT0019043 | 61.6 | 36.5 | 0.59 | -0.75 |
| hsa-miR-2116* | MIMAT0011161 | 31.2 | 36.4 | 1.17 | 0.22 |
| hsa-miR-93 | MIMAT0000093 | 48.0 | 36.2 | 0.75 | -0.41 |
| hsa-miR-4664-3p | MIMAT0019738 | 39.2 | 35.9 | 0.91 | -0.13 |
| hsa-miR-4446-3p | MIMAT0018965 | 44.5 | 35.5 | 0.80 | -0.33 |
| hsa-miR-4639-3p | MIMAT0019698 | 26.3 | 35.3 | 1.35 | 0.43 |
| hsa-miR-591 | MIMAT0003259 | 9.6 | 34.8 | 3.62 | 1.86 |
| hsa-miR-3675-3p | MIMAT0018099 | 37.8 | 34.7 | 0.92 | -0.12 |
| hsa-miR-3176 | MIMAT0015053 | 27.8 | 34.4 | 1.24 | 0.31 |
| hsa-miR-943 | MIMAT0004986 | 34.3 | 34.4 | 1.00 | 0.00 |
| hsa-miR-320b | MIMAT0005792 | 49.9 | 34.3 | 0.69 | -0.54 |
| hsa-miR-4756-5p | MIMAT0019899 | 48.5 | 34.3 | 0.71 | -0.50 |
| hsa-miR-4717-5p | MIMAT0019829 | 18.9 | 34.2 | 1.81 | 0.86 |
| hsa-miR-4419a | MIMAT0018931 | 23.0 | 34.1 | 1.48 | 0.57 |
| hsa-miR-4722-5p | MIMAT0019836 | 51.9 | 34.0 | 0.66 | -0.61 |
| hsa-miR-3667-3p | MIMAT0018090 | 37.5 | 34.0 | 0.91 | -0.14 |
| hsa-miR-26b* | MIMAT0004500 | 36.7 | 33.8 | 0.92 | -0.12 |
| hsa-miR-2467-3p | MIMAT0019953 | 21.8 | 33.4 | 1.53 | 0.62 |
| hsa-miR-885-3p | MIMAT0004948 | 22.8 | 33.2 | 1.46 | 0.54 |
| hsa-miR-4312 | MIMAT0016864 | 34.4 | 33.2 | 0.96 | -0.05 |
| hsa-miR-27a | MIMAT0000084 | 46.6 | 33.2 | 0.71 | -0.49 |
| hsa-miR-4481 | MIMAT0019015 | 40.0 | 33.1 | 0.83 | -0.27 |
| hsa-miR-4462 | MIMAT0018986 | 27.3 | 33.0 | 1.21 | 0.27 |
| hsa-miR-4296 | MIMAT0016845 | 11.5 | 32.9 | 2.86 | 1.52 |
| hsa-miR-1266 | MIMAT0005920 | 27.2 | 32.8 | 1.21 | 0.27 |
| hsa-miR-4751 | MIMAT0019888 | 33.9 | 32.8 | 0.97 | -0.05 |
| hsa-miR-3189-5p | MIMAT0019217 | 32.0 | 32.8 | 1.02 | 0.04 |
| hsa-miR-4483 | MIMAT0019017 | 25.6 | 32.7 | 1.28 | 0.35 |
| hsa-miR-485-3p | MIMAT0002176 | 51.6 | 32.7 | 0.63 | -0.66 |
| hsa-miR-4326 | MIMAT0016888 | 35.1 | 32.6 | 0.93 | -0.11 |
| hsa-miR-3147 | MIMAT0015019 | 36.1 | 32.4 | 0.90 | -0.15 |
| hsa-miR-3972 | MIMAT0019357 | 14.0 | 32.0 | 2.28 | 1.19 |
| hsa-miR-650 | MIMAT0003320 | 11.0 | 31.6 | 2.87 | 1.52 |
| hsa-miR-125a-3p | MIMAT0004602 | 27.4 | 31.6 | 1.15 | 0.20 |
| hsa-miR-181b | MIMAT0000257 | 26.0 | 31.4 | 1.21 | 0.27 |
| hsa-miR-892b | MIMAT0004918 | 28.4 | 31.3 | 1.10 | 0.14 |
| hsa-miR-214 | MIMAT0000271 | 36.1 | 31.1 | 0.86 | -0.21 |
| hsa-miR-4673 | MIMAT0019755 | 32.3 | 31.0 | 0.96 | -0.06 |
| hsa-miR-4664-5p | MIMAT0019737 | 48.9 | 31.0 | 0.63 | -0.66 |
| hsa-miR-4804-3p | MIMAT0019985 | 24.8 | 30.8 | 1.24 | 0.31 |
| hsa-miR-939 | MIMAT0004982 | 69.2 | 30.7 | 0.44 | -1.17 |
| hsa-miR-2113 | MIMAT0009206 | 16.2 | 30.7 | 1.89 | 0.92 |
| hsa-miR-4540 | MIMAT0019083 | 20.6 | 30.6 | 1.49 | 0.57 |
| hsa-miR-3191 | MIMAT0015075 | 23.7 | 30.6 | 1.29 | 0.37 |
| hsa-miR-4259 | MIMAT0016880 | 30.9 | 30.6 | 0.99 | -0.01 |
| hsa-miR-3194-5p | MIMAT0015078 | 49.1 | 30.6 | 0.62 | -0.68 |
| hsa-miR-425* | MIMAT0001343 | 15.9 | 30.4 | 1.92 | 0.94 |
| hsa-miR-3158-3p | MIMAT0015032 | 20.8 | 30.3 | 1.46 | 0.54 |
| hsa-miR-99b | MIMAT0000689 | 27.6 | 30.2 | 1.10 | 0.13 |
| hsa-miR-4472 | MIMAT0018999 | 28.4 | 30.1 | 1.06 | 0.08 |
| hsa-let-7b | MIMAT0000063 | 47.8 | 30.0 | 0.63 | -0.67 |
| hsa-miR-432* | MIMAT0002815 | 26.8 | 29.9 | 1.12 | 0.16 |
| hsa-miR-3187-5p | MIMAT0019216 | 23.8 | 29.7 | 1.25 | 0.32 |
| hsa-miR-4710 | MIMAT0019815 | 15.1 | 29.7 | 1.96 | 0.97 |
| hsa-miR-3120-5p | MIMAT0019198 | 22.2 | 29.6 | 1.33 | 0.42 |
| hsa-miR-3180-5p | MIMAT0015057 | 31.2 | 29.5 | 0.95 | -0.08 |
| hsa-miR-3676 | MIMAT0018100 | 9.4 | 29.3 | 3.12 | 1.64 |
| hsa-miR-345 | MIMAT0000772 | 19.5 | 29.2 | 1.49 | 0.58 |
| hsa-miR-4641 | MIMAT0019701 | 14.8 | 29.1 | 1.97 | 0.98 |
| hsa-miR-593 | MIMAT0004802 | 16.8 | 29.1 | 1.73 | 0.79 |
| hsa-miR-3944-5p | MIMAT0019231 | 35.2 | 29.0 | 0.82 | -0.28 |
| hsa-miR-3678-3p | MIMAT0018103 | 54.3 | 29.0 | 0.53 | -0.91 |
| hsa-miR-1470 | MIMAT0007348 | 31.0 | 28.9 | 0.93 | -0.10 |
| hsa-miR-4299 | MIMAT0016851 | 38.7 | 28.8 | 0.74 | -0.43 |
| hsa-miR-634 | MIMAT0003304 | 20.5 | 28.8 | 1.40 | 0.49 |
| hsa-miR-4474-3p | MIMAT0019001 | 22.8 | 28.8 | 1.26 | 0.34 |
| hsa-miR-1247 | MIMAT0005899 | 14.6 | 28.7 | 1.97 | 0.98 |
| hsa-miR-15b | MIMAT0000417 | 59.6 | 28.6 | 0.48 | -1.06 |
| hsa-miR-874 | MIMAT0004911 | 33.4 | 28.6 | 0.86 | -0.22 |
| hsa-miR-4708-5p | MIMAT0019809 | 37.9 | 28.6 | 0.76 | -0.40 |
| hsa-miR-4324 | MIMAT0016876 | 49.2 | 28.5 | 0.58 | -0.79 |
| hsa-miR-4520a-5p, hsa-miR-4520b-5p | MIMAT0019235, MIMAT0020299 | 9.0 | 28.3 | 3.14 | 1.65 |
| hsa-miR-342-3p | MIMAT0000753 | 27.1 | 28.2 | 1.04 | 0.06 |
| hsa-miR-4653-5p | MIMAT0019718 | 23.4 | 28.2 | 1.20 | 0.27 |
| hsa-miR-4448 | MIMAT0018967 | 40.5 | 28.1 | 0.69 | -0.53 |
| hsa-let-7f | MIMAT0000067 | 53.5 | 28.0 | 0.52 | -0.93 |
| hsa-miR-661 | MIMAT0003324 | 20.3 | 28.0 | 1.38 | 0.47 |
| hsa-miR-3922-5p | MIMAT0019227 | 23.8 | 28.0 | 1.18 | 0.24 |
| hsa-miR-3943 | MIMAT0018359 | 14.7 | 27.9 | 1.90 | 0.93 |
| hsa-miR-4743 | MIMAT0019874 | 28.2 | 27.8 | 0.99 | -0.02 |
| hsa-miR-3714 | MIMAT0018165 | 31.0 | 27.8 | 0.89 | -0.16 |
| hsa-miR-4686 | MIMAT0019773 | 20.7 | 27.7 | 1.34 | 0.42 |
| hsa-miR-512-5p | MIMAT0002822 | 29.0 | 27.6 | 0.95 | -0.07 |
| hsa-miR-4736 | MIMAT0019862 | 21.4 | 27.6 | 1.29 | 0.37 |
| hsa-miR-25 | MIMAT0000081 | 34.5 | 27.5 | 0.80 | -0.33 |
| hsa-miR-4329 | MIMAT0016923 | 22.6 | 27.5 | 1.22 | 0.28 |
| hsa-miR-4478 | MIMAT0019006 | 47.9 | 27.4 | 0.57 | -0.81 |
| hsa-miR-506 | MIMAT0002878 | 18.0 | 27.3 | 1.52 | 0.60 |
| hsa-miR-4267 | MIMAT0016893 | 14.3 | 27.0 | 1.89 | 0.92 |
| hsa-miR-1254 | MIMAT0005905 | 21.3 | 27.0 | 1.27 | 0.34 |
| hsa-miR-4754 | MIMAT0019894 | 14.2 | 26.8 | 1.88 | 0.91 |
| hsa-miR-4800-3p | MIMAT0019979 | 45.7 | 26.8 | 0.59 | -0.77 |
| hsa-miR-1193 | MIMAT0015049 | 19.9 | 26.7 | 1.34 | 0.42 |
| hsa-miR-550b | MIMAT0018445 | 24.6 | 26.5 | 1.08 | 0.11 |
| hsa-miR-1231 | MIMAT0005586 | 30.7 | 26.5 | 0.86 | -0.21 |
| hsa-miR-3144-3p | MIMAT0015015 | 13.5 | 26.3 | 1.96 | 0.97 |
| hsa-miR-1273f | MIMAT0020601 | 23.9 | 26.2 | 1.10 | 0.13 |
| hsa-miR-632 | MIMAT0003302 | 13.3 | 26.2 | 1.96 | 0.97 |
| hsa-miR-154 | MIMAT0000452 |  | 26.1 |  |  |
| hsa-miR-1288 | MIMAT0005942 | 26.1 | 26.1 | 1.00 | 0.00 |
| hsa-miR-371b-5p | MIMAT0019892 | 29.3 | 26.0 | 0.89 | -0.17 |
| hsa-miR-4640-3p | MIMAT0019700 | 19.0 | 25.8 | 1.36 | 0.44 |
| hsa-miR-4260 | MIMAT0016881 | 10.3 | 25.6 | 2.48 | 1.31 |
| hsa-miR-204 | MIMAT0000265 |  | 25.6 |  |  |
| hsa-let-7a-2* | MIMAT0010195 | 26.7 | 25.6 | 0.96 | -0.06 |
| hsa-miR-181a-2* | MIMAT0004558 |  | 25.6 |  |  |
| hsa-miR-125a-5p | MIMAT0000443 | 35.1 | 25.5 | 0.73 | -0.46 |
| hsa-miR-4701-3p | MIMAT0019799 | 19.7 | 25.5 | 1.29 | 0.37 |
| hsa-miR-4701-5p | MIMAT0019798 | 19.7 | 25.4 | 1.29 | 0.36 |
| hsa-miR-4700-5p | MIMAT0019796 | 26.1 | 25.3 | 0.97 | -0.05 |
| hsa-miR-361-3p | MIMAT0004682 | 23.4 | 25.3 | 1.08 | 0.11 |
| hsa-miR-4746-3p | MIMAT0019881 | 32.2 | 25.3 | 0.78 | -0.35 |
| hsa-miR-1914 | MIMAT0007889 | 31.5 | 25.1 | 0.80 | -0.33 |
| hsa-miR-324-3p | MIMAT0000762 | 13.5 | 25.0 | 1.86 | 0.89 |
| hsa-miR-4776-5p | MIMAT0019932 | 28.5 | 25.0 | 0.88 | -0.19 |
| hsa-miR-4446-5p | MIMAT0019233 | 19.6 | 24.9 | 1.27 | 0.35 |
| hsa-miR-519d | MIMAT0002853 | 18.1 | 24.9 | 1.37 | 0.46 |
| hsa-miR-210 | MIMAT0000267 | 17.1 | 24.8 | 1.45 | 0.54 |
| hsa-miR-339-5p | MIMAT0000764 | 35.6 | 24.8 | 0.70 | -0.52 |
| hsa-miR-4529-5p | MIMAT0019236 | 13.5 | 24.8 | 1.83 | 0.87 |
| hsa-miR-937 | MIMAT0004980 | 29.8 | 24.5 | 0.82 | -0.28 |
| hsa-miR-659 | MIMAT0003337 |  | 24.5 |  |  |
| hsa-miR-501-5p | MIMAT0002872 |  | 24.5 |  |  |
| hsa-let-7d* | MIMAT0004484 | 27.2 | 24.5 | 0.90 | -0.15 |
| hsa-miR-665 | MIMAT0004952 | 28.2 | 24.4 | 0.87 | -0.21 |
| hsa-miR-3692* | MIMAT0018121 | 23.3 | 24.4 | 1.05 | 0.06 |
| hsa-miR-4691-5p | MIMAT0019781 | 32.2 | 24.4 | 0.76 | -0.40 |
| hsa-miR-3126-5p | MIMAT0014989 | 28.5 | 24.3 | 0.85 | -0.23 |
| hsa-miR-498 | MIMAT0002824 | 9.2 | 24.2 | 2.63 | 1.40 |
| hsa-miR-3935 | MIMAT0018350 | 23.1 | 24.2 | 1.05 | 0.07 |
| hsa-miR-664 | MIMAT0005949 | 18.2 | 24.1 | 1.32 | 0.40 |
| hsa-let-7b* | MIMAT0004482 | 38.7 | 24.0 | 0.62 | -0.69 |
| hsa-miR-4747-3p | MIMAT0019883 | 23.6 | 23.9 | 1.02 | 0.02 |
| hsa-miR-4800-5p | MIMAT0019978 | 29.9 | 23.9 | 0.80 | -0.32 |
| hsa-miR-214* | MIMAT0004564 | 14.8 | 23.7 | 1.61 | 0.69 |
| hsa-miR-3187-3p | MIMAT0015069 | 24.5 | 23.7 | 0.97 | -0.05 |
| hsa-miR-191 | MIMAT0000440 | 32.7 | 23.6 | 0.72 | -0.47 |
| hsa-miR-133b | MIMAT0000770 | 17.8 | 23.6 | 1.32 | 0.40 |
| hsa-miR-200c | MIMAT0000617 | 18.3 | 23.5 | 1.29 | 0.37 |
| hsa-miR-4419b | MIMAT0019034 | 45.9 | 23.5 | 0.51 | -0.96 |
| hsa-miR-26a | MIMAT0000082 | 34.6 | 23.4 | 0.68 | -0.57 |
| hsa-miR-4254 | MIMAT0016884 | 20.9 | 23.4 | 1.12 | 0.16 |
| hsa-miR-4638-3p | MIMAT0019696 | 30.9 | 23.3 | 0.76 | -0.40 |
| hsa-miR-141* | MIMAT0004598 |  | 23.3 |  |  |
| hsa-miR-151-5p | MIMAT0004697 | 15.4 | 23.3 | 1.51 | 0.59 |
| hsa-miR-466 | MIMAT0015002 | 32.3 | 23.3 | 0.72 | -0.47 |
| hsa-miR-34b | MIMAT0004676 | 11.0 | 23.3 | 2.11 | 1.08 |
| hsa-miR-212 | MIMAT0000269 | 17.5 | 23.3 | 1.33 | 0.41 |
| hsa-miR-4783-3p | MIMAT0019947 | 30.4 | 23.3 | 0.77 | -0.39 |
| hsa-miR-4725-5p | MIMAT0019843 | 37.8 | 23.0 | 0.61 | -0.72 |
| hsa-miR-320c | MIMAT0005793 | 46.1 | 23.0 | 0.50 | -1.00 |
| hsa-miR-4786-3p | MIMAT0019955 | 10.6 | 22.8 | 2.15 | 1.10 |
| hsa-miR-1261 | MIMAT0005913 | 8.6 | 22.7 | 2.63 | 1.40 |
| hsa-miR-199a-3p, hsa-miR-199b-3p | MIMAT0000232, MIMAT0004563 | 17.3 | 22.7 | 1.31 | 0.39 |
| hsa-miR-134 | MIMAT0000447 | 27.9 | 22.7 | 0.81 | -0.30 |
| hsa-miR-211 | MIMAT0000268 | 41.9 | 22.6 | 0.54 | -0.89 |
| hsa-miR-451 | MIMAT0001631 | 29.8 | 22.5 | 0.76 | -0.40 |
| hsa-miR-493 | MIMAT0003161 | 14.3 | 22.5 | 1.58 | 0.66 |
| hsa-miR-3622b-5p | MIMAT0018005 | 35.8 | 22.5 | 0.63 | -0.67 |
| hsa-miR-4525 | MIMAT0019064 | 31.2 | 22.5 | 0.72 | -0.47 |
| hsa-miR-378i | MIMAT0019074 | 18.2 | 22.5 | 1.24 | 0.31 |
| hsa-miR-34a | MIMAT0000255 | 16.4 | 22.5 | 1.37 | 0.46 |
| hsa-miR-935 | MIMAT0004978 | 23.0 | 22.5 | 0.98 | -0.04 |
| hsa-miR-296-3p | MIMAT0004679 | 14.3 | 22.4 | 1.57 | 0.65 |
| hsa-miR-4274 | MIMAT0016906 | 37.2 | 22.4 | 0.60 | -0.73 |
| hsa-miR-3065-3p | MIMAT0015378 | 9.9 | 22.4 | 2.26 | 1.18 |
| hsa-miR-184 | MIMAT0000454 | 9.9 | 22.3 | 2.25 | 1.17 |
| hsa-miR-4305 | MIMAT0016857 | 28.7 | 22.3 | 0.78 | -0.37 |
| hsa-miR-4738-3p | MIMAT0019867 |  | 22.2 |  |  |
| hsa-miR-4456 | MIMAT0018978 | 10.7 | 22.2 | 2.07 | 1.05 |
| hsa-miR-146b-3p | MIMAT0004766 | 19.7 | 22.1 | 1.12 | 0.17 |
| hsa-miR-409-3p | MIMAT0001639 | 11.3 | 22.0 | 1.94 | 0.96 |
| hsa-miR-3127-5p | MIMAT0014990 | 16.7 | 22.0 | 1.32 | 0.40 |
| hsa-miR-1322 | MIMAT0005953 | 14.5 | 21.9 | 1.51 | 0.60 |
| hsa-miR-4749-3p | MIMAT0019886 | 29.6 | 21.9 | 0.74 | -0.44 |
| hsa-miR-4780 | MIMAT0019939 | 21.1 | 21.7 | 1.03 | 0.04 |
| hsa-miR-4746-5p | MIMAT0019880 | 13.7 | 21.7 | 1.59 | 0.67 |
| hsa-let-7f-1* | MIMAT0004486 | 27.9 | 21.6 | 0.78 | -0.37 |
| hsa-miR-3934 | MIMAT0018349 | 30.7 | 21.4 | 0.70 | -0.52 |
| hsa-miR-3160-5p | MIMAT0019212 | 12.2 | 21.4 | 1.75 | 0.81 |
| hsa-miR-30e | MIMAT0000692 |  | 21.4 |  |  |
| hsa-miR-4470 | MIMAT0018997 | 19.7 | 21.3 | 1.08 | 0.11 |
| hsa-miR-4319 | MIMAT0016870 | 15.9 | 21.2 | 1.34 | 0.42 |
| hsa-miR-4711-3p | MIMAT0019817 | 18.0 | 21.2 | 1.18 | 0.24 |
| hsa-miR-595 | MIMAT0003263 | 18.9 | 21.2 | 1.12 | 0.16 |
| hsa-miR-1915* | MIMAT0007891 | 22.2 | 21.1 | 0.95 | -0.07 |
| hsa-miR-30a | MIMAT0000087 | 28.3 | 21.1 | 0.75 | -0.42 |
| hsa-miR-744* | MIMAT0004946 |  | 21.1 |  |  |
| hsa-miR-4768-5p | MIMAT0019920 |  | 21.1 |  |  |
| hsa-let-7i | MIMAT0000415 | 76.6 | 21.0 | 0.27 | -1.87 |
| hsa-miR-4646-5p | MIMAT0019707 | 25.8 | 21.0 | 0.81 | -0.30 |
| hsa-miR-3976 | MIMAT0019361 |  | 20.9 |  |  |
| hsa-miR-4436b-3p | MIMAT0019941 | 17.6 | 20.9 | 1.19 | 0.25 |
| hsa-miR-3162-3p | MIMAT0019213 | 20.4 | 20.8 | 1.02 | 0.03 |
| hsa-miR-4727-3p | MIMAT0019848 | 16.8 | 20.8 | 1.23 | 0.30 |
| hsa-miR-103a-2* | MIMAT0009196 |  | 20.7 |  |  |
| hsa-miR-4436a | MIMAT0018952 | 12.7 | 20.6 | 1.62 | 0.70 |
| hsa-miR-185* | MIMAT0004611 | 19.5 | 20.6 | 1.06 | 0.08 |
| hsa-miR-4506 | MIMAT0019042 | 27.8 | 20.6 | 0.74 | -0.43 |
| hsa-miR-3689d | MIMAT0019008 |  | 20.6 |  |  |
| hsa-miR-199b-5p | MIMAT0000263 | 10.1 | 20.5 | 2.04 | 1.03 |
| hsa-miR-25* | MIMAT0004498 | 9.1 | 20.3 | 2.23 | 1.16 |
| hsa-miR-654-3p | MIMAT0004814 | 23.2 | 20.3 | 0.87 | -0.20 |
| hsa-miR-2355-5p | MIMAT0016895 | 16.9 | 20.2 | 1.20 | 0.26 |
| hsa-miR-3200-3p | MIMAT0015085 |  | 20.2 |  |  |
| hsa-miR-4647 | MIMAT0019709 |  | 20.1 |  |  |
| hsa-miR-4756-3p | MIMAT0019900 | 17.7 | 20.0 | 1.13 | 0.17 |
| hsa-miR-140-5p | MIMAT0000431 | 23.6 | 20.0 | 0.85 | -0.24 |
| hsa-miR-140-3p | MIMAT0004597 | 31.4 | 19.8 | 0.63 | -0.66 |
| hsa-miR-4421 | MIMAT0018934 | 8.6 | 19.7 | 2.28 | 1.19 |
| hsa-miR-3198 | MIMAT0015083 | 17.0 | 19.6 | 1.15 | 0.21 |
| hsa-miR-30c-1* | MIMAT0004674 | 36.5 | 19.6 | 0.54 | -0.90 |
| hsa-miR-3155b | MIMAT0019012 | 10.6 | 19.5 | 1.84 | 0.88 |
| hsa-miR-4502 | MIMAT0019038 | 41.6 | 19.4 | 0.47 | -1.10 |
| hsa-miR-572 | MIMAT0003237 |  | 19.4 |  |  |
| hsa-miR-4437 | MIMAT0018953 | 9.5 | 19.3 | 2.04 | 1.03 |
| hsa-miR-4767 | MIMAT0019919 | 22.5 | 19.3 | 0.86 | -0.22 |
| hsa-miR-1267 | MIMAT0005921 |  | 19.3 |  |  |
| hsa-miR-3186-5p | MIMAT0015067 | 21.8 | 19.2 | 0.88 | -0.18 |
| hsa-miR-517c | MIMAT0002866 |  | 19.1 |  |  |
| hsa-miR-1972 | MIMAT0009447 | 11.9 | 19.0 | 1.60 | 0.67 |
| hsa-miR-4747-5p | MIMAT0019882 | 20.8 | 18.9 | 0.91 | -0.13 |
| hsa-miR-769-3p | MIMAT0003887 | 10.8 | 18.8 | 1.74 | 0.80 |
| hsa-miR-4682 | MIMAT0019767 | 34.0 | 18.8 | 0.55 | -0.85 |
| hsa-miR-124 | MIMAT0000422 |  | 18.8 |  |  |
| hsa-miR-3650 | MIMAT0018070 | 17.6 | 18.7 | 1.06 | 0.09 |
| hsa-miR-516b | MIMAT0002859 |  | 18.6 |  |  |
| hsa-miR-3122 | MIMAT0014984 | 8.7 | 18.6 | 2.13 | 1.09 |
| hsa-miR-372 | MIMAT0000724 |  | 18.6 |  |  |
| hsa-miR-3150b-3p | MIMAT0018194 | 15.0 | 18.5 | 1.23 | 0.30 |
| hsa-miR-623 | MIMAT0003292 |  | 18.5 |  |  |
| hsa-miR-551b* | MIMAT0004794 | 10.7 | 18.4 | 1.72 | 0.79 |
| hsa-miR-4251 | MIMAT0016883 | 13.6 | 18.4 | 1.35 | 0.43 |
| hsa-miR-205 | MIMAT0000266 | 12.5 | 18.3 | 1.47 | 0.55 |
| hsa-miR-608 | MIMAT0003276 | 10.7 | 18.2 | 1.71 | 0.77 |
| hsa-miR-378g | MIMAT0018937 | 19.2 | 18.2 | 0.95 | -0.08 |
| hsa-miR-18b* | MIMAT0004751 | 27.3 | 18.2 | 0.67 | -0.58 |
| hsa-miR-1306 | MIMAT0005950 | 13.4 | 18.1 | 1.35 | 0.43 |
| hsa-miR-15b* | MIMAT0004586 |  | 18.1 |  |  |
| hsa-miR-512-3p | MIMAT0002823 | 16.3 | 18.0 | 1.10 | 0.14 |
| hsa-miR-378 | MIMAT0000732 | 14.6 | 17.9 | 1.23 | 0.30 |
| hsa-miR-4489 | MIMAT0019023 | 19.6 | 17.9 | 0.91 | -0.13 |
| hsa-let-7e | MIMAT0000066 |  | 17.8 |  |  |
| hsa-miR-18a* | MIMAT0002891 | 22.8 | 17.7 | 0.78 | -0.36 |
| hsa-miR-376c | MIMAT0000720 |  | 17.7 |  |  |
| hsa-miR-4649-3p | MIMAT0019712 | 43.3 | 17.6 | 0.41 | -1.30 |
| hsa-miR-654-5p | MIMAT0003330 | 12.9 | 17.6 | 1.36 | 0.45 |
| hsa-miR-330-3p | MIMAT0000751 | 13.3 | 17.6 | 1.32 | 0.40 |
| hsa-miR-18b | MIMAT0001412 | 16.8 | 17.6 | 1.05 | 0.07 |
| hsa-miR-299-5p | MIMAT0002890 | 14.9 | 17.5 | 1.18 | 0.23 |
| hsa-miR-4317 | MIMAT0016872 |  | 17.4 |  |  |
| hsa-miR-3925-5p | MIMAT0018200 | 24.3 | 17.3 | 0.71 | -0.49 |
| hsa-miR-23c | MIMAT0018000 | 8.7 | 17.3 | 1.98 | 0.99 |
| hsa-miR-517b | MIMAT0002857 |  | 17.2 |  |  |
| hsa-miR-4533 | MIMAT0019072 | 21.4 | 17.2 | 0.81 | -0.31 |
| hsa-miR-551a | MIMAT0003214 | 15.2 | 17.2 | 1.13 | 0.18 |
| hsa-miR-4644 | MIMAT0019704 |  | 17.1 |  |  |
| hsa-miR-138 | MIMAT0000430 | 11.7 | 17.1 | 1.46 | 0.55 |
| hsa-miR-1204 | MIMAT0005868 | 26.6 | 17.1 | 0.64 | -0.64 |
| hsa-miR-323-3p | MIMAT0000755 | 8.7 | 17.0 | 1.97 | 0.98 |
| hsa-miR-3591-3p | MIMAT0019877 | 17.4 | 17.0 | 0.98 | -0.03 |
| hsa-miR-4652-5p | MIMAT0019716 |  | 17.0 |  |  |
| hsa-miR-4740-5p | MIMAT0019869 | 9.0 | 16.9 | 1.88 | 0.91 |
| hsa-miR-3189-3p | MIMAT0015071 |  | 16.9 |  |  |
| hsa-miR-4487 | MIMAT0019021 | 30.2 | 16.8 | 0.55 | -0.85 |
| hsa-miR-631 | MIMAT0003300 | 25.6 | 16.7 | 0.65 | -0.61 |
| hsa-miR-4653-3p | MIMAT0019719 | 17.1 | 16.7 | 0.98 | -0.03 |
| hsa-miR-4263 | MIMAT0016898 | 8.6 | 16.7 | 1.96 | 0.97 |
| hsa-miR-301a | MIMAT0000688 |  | 16.7 |  |  |
| hsa-let-7g* | MIMAT0004584 |  | 16.6 |  |  |
| hsa-miR-181c | MIMAT0000258 |  | 16.6 |  |  |
| hsa-miR-1269b | MIMAT0019059 | 11.2 | 16.6 | 1.48 | 0.57 |
| hsa-miR-4465 | MIMAT0018992 | 30.3 | 16.6 | 0.55 | -0.87 |
| hsa-miR-330-5p | MIMAT0004693 | 11.6 | 16.5 | 1.41 | 0.50 |
| hsa-miR-206 | MIMAT0000462 | 9.1 | 16.4 | 1.81 | 0.85 |
| hsa-miR-193b* | MIMAT0004767 | 15.6 | 16.4 | 1.05 | 0.07 |
| hsa-miR-548ab | MIMAT0018928 | 20.7 | 16.3 | 0.79 | -0.34 |
| hsa-miR-3669 | MIMAT0018092 |  | 16.3 |  |  |
| hsa-miR-4712-5p | MIMAT0019818 | 8.7 | 16.3 | 1.88 | 0.91 |
| hsa-miR-4496 | MIMAT0019031 | 18.0 | 16.3 | 0.90 | -0.15 |
| hsa-miR-3064-5p | MIMAT0019864 | 11.2 | 16.3 | 1.45 | 0.53 |
| hsa-miR-431 | MIMAT0001625 |  | 16.2 |  |  |
| hsa-miR-4789-5p | MIMAT0019959 |  | 16.2 |  |  |
| hsa-miR-361-5p | MIMAT0000703 |  | 16.2 |  |  |
| hsa-miR-30c | MIMAT0000244 | 21.7 | 16.2 | 0.74 | -0.43 |
| hsa-miR-1273d | MIMAT0015090 |  | 16.1 |  |  |
| hsa-miR-4330 | MIMAT0016924 | 21.7 | 16.1 | 0.74 | -0.43 |
| hsa-miR-3691-3p | MIMAT0019224 | 17.8 | 16.0 | 0.90 | -0.15 |
| hsa-miR-877 | MIMAT0004949 | 14.6 | 15.9 | 1.09 | 0.13 |
| hsa-miR-2276 | MIMAT0011775 | 14.8 | 15.9 | 1.07 | 0.10 |
| hsa-miR-492 | MIMAT0002812 | 15.1 | 15.9 | 1.05 | 0.07 |
| hsa-miR-30b | MIMAT0000420 | 34.0 | 15.8 | 0.47 | -1.10 |
| hsa-miR-320e | MIMAT0015072 | 15.7 | 15.8 | 1.00 | 0.01 |
| hsa-miR-5096 | MIMAT0020603 | 14.5 | 15.8 | 1.09 | 0.12 |
| hsa-miR-1293 | MIMAT0005883 | 16.7 | 15.8 | 0.94 | -0.08 |
| hsa-miR-4261 | MIMAT0016890 | 16.7 | 15.7 | 0.94 | -0.08 |
| hsa-miR-550a* | MIMAT0003257 | 15.7 | 15.7 | 1.00 | 0.00 |
| hsa-miR-194* | MIMAT0004671 | 12.9 | 15.7 | 1.22 | 0.29 |
| hsa-miR-423-3p | MIMAT0001340 | 13.0 | 15.7 | 1.21 | 0.27 |
| hsa-miR-4518 | MIMAT0019055 | 17.1 | 15.7 | 0.92 | -0.12 |
| hsa-miR-517* | MIMAT0002851 | 11.9 | 15.6 | 1.31 | 0.39 |
| hsa-miR-99b* | MIMAT0004678 | 16.6 | 15.6 | 0.94 | -0.09 |
| hsa-miR-4538 | MIMAT0019081 | 25.0 | 15.5 | 0.62 | -0.69 |
| hsa-miR-3173-3p | MIMAT0015048 | 8.6 | 15.5 | 1.79 | 0.84 |
| hsa-miR-187 | MIMAT0000262 | 31.7 | 15.5 | 0.49 | -1.04 |
| hsa-miR-4684-3p | MIMAT0019770 | 10.7 | 15.5 | 1.44 | 0.53 |
| hsa-miR-1303 | MIMAT0005891 | 10.6 | 15.4 | 1.45 | 0.54 |
| hsa-miR-593* | MIMAT0003261 | 31.5 | 15.4 | 0.49 | -1.04 |
| hsa-miR-338-3p | MIMAT0000763 |  | 15.4 |  |  |
| hsa-miR-3126-3p | MIMAT0015377 | 16.8 | 15.4 | 0.91 | -0.13 |
| hsa-miR-200a* | MIMAT0001620 |  | 15.3 |  |  |
| hsa-miR-190 | MIMAT0000458 |  | 15.3 |  |  |
| hsa-miR-875-3p | MIMAT0004923 | 12.0 | 15.2 | 1.27 | 0.35 |
| hsa-miR-3174 | MIMAT0015051 |  | 15.2 |  |  |
| hsa-miR-767-3p | MIMAT0003883 | 19.0 | 15.2 | 0.80 | -0.32 |
| hsa-miR-34b* | MIMAT0000685 |  | 15.2 |  |  |
| hsa-miR-4287 | MIMAT0016917 | 26.8 | 15.1 | 0.57 | -0.82 |
| hsa-miR-298 | MIMAT0004901 | 10.1 | 15.1 | 1.51 | 0.59 |
| hsa-miR-4717-3p | MIMAT0019830 | 11.9 | 15.1 | 1.26 | 0.34 |
| hsa-miR-548q | MIMAT0011163 | 18.7 | 15.1 | 0.80 | -0.31 |
| hsa-miR-1291 | MIMAT0005881 | 8.6 | 15.1 | 1.76 | 0.81 |
| hsa-miR-4654 | MIMAT0019720 | 18.4 | 15.0 | 0.82 | -0.29 |
| hsa-miR-195* | MIMAT0004615 |  | 15.0 |  |  |
| hsa-miR-449a | MIMAT0001541 | 18.3 | 15.0 | 0.82 | -0.29 |
| hsa-miR-500a | MIMAT0004773 |  | 14.9 |  |  |
| hsa-miR-3975 | MIMAT0019360 |  | 14.9 |  |  |
| hsa-miR-300 | MIMAT0004903 |  | 14.9 |  |  |
| hsa-miR-488* | MIMAT0002804 | 17.2 | 14.9 | 0.86 | -0.21 |
| hsa-miR-4776-3p | MIMAT0019933 | 18.9 | 14.8 | 0.78 | -0.35 |
| hsa-miR-224* | MIMAT0009198 | 12.5 | 14.8 | 1.18 | 0.24 |
| hsa-miR-505* | MIMAT0004776 |  | 14.8 |  |  |
| hsa-miR-3619-5p | MIMAT0017999 | 22.5 | 14.7 | 0.66 | -0.61 |
| hsa-miR-363* | MIMAT0003385 |  | 14.7 |  |  |
| hsa-miR-3911 | MIMAT0018185 | 12.5 | 14.7 | 1.18 | 0.23 |
| hsa-miR-2909 | MIMAT0013863 | 15.1 | 14.7 | 0.97 | -0.04 |
| hsa-miR-936 | MIMAT0004979 | 12.6 | 14.7 | 1.16 | 0.22 |
| hsa-miR-196b | MIMAT0001080 | 11.9 | 14.6 | 1.22 | 0.29 |
| hsa-miR-4311 | MIMAT0016863 |  | 14.6 |  |  |
| hsa-miR-4473 | MIMAT0019000 | 11.4 | 14.6 | 1.28 | 0.36 |
| hsa-miR-3144-5p | MIMAT0015014 |  | 14.6 |  |  |
| hsa-miR-4669 | MIMAT0019749 | 15.2 | 14.5 | 0.96 | -0.07 |
| hsa-miR-4761-5p | MIMAT0019908 |  | 14.5 |  |  |
| hsa-miR-4738-5p | MIMAT0019866 |  | 14.5 |  |  |
| hsa-miR-3655 | MIMAT0018075 | 12.3 | 14.5 | 1.17 | 0.23 |
| hsa-miR-4420 | MIMAT0018933 | 11.1 | 14.5 | 1.30 | 0.38 |
| hsa-miR-4444 | MIMAT0018962 |  | 14.5 |  |  |
| hsa-miR-3687 | MIMAT0018115 | 18.7 | 14.5 | 0.77 | -0.37 |
| hsa-miR-4676-5p | MIMAT0019758 | 27.9 | 14.4 | 0.52 | -0.95 |
| hsa-miR-1273e | MIMAT0018079 | 17.8 | 14.4 | 0.81 | -0.31 |
| hsa-miR-128 | MIMAT0000424 |  | 14.4 |  |  |
| hsa-miR-378e | MIMAT0018927 |  | 14.3 |  |  |
| hsa-miR-22* | MIMAT0004495 | 10.9 | 14.3 | 1.31 | 0.39 |
| hsa-miR-4714-5p | MIMAT0019822 | 27.5 | 14.2 | 0.52 | -0.95 |
| hsa-miR-3150a-5p | MIMAT0019206 | 9.2 | 14.2 | 1.54 | 0.62 |
| hsa-miR-186 | MIMAT0000456 |  | 14.2 |  |  |
| hsa-miR-1227 | MIMAT0005580 | 12.5 | 14.2 | 1.13 | 0.18 |
| hsa-miR-4522 | MIMAT0019060 | 11.4 | 14.1 | 1.24 | 0.31 |
| hsa-miR-619 | MIMAT0003288 |  | 14.1 |  |  |
| hsa-miR-1207-3p | MIMAT0005872 | 23.4 | 14.1 | 0.60 | -0.73 |
| hsa-miR-487b | MIMAT0003180 |  | 14.1 |  |  |
| hsa-miR-338-5p | MIMAT0004701 |  | 14.0 |  |  |
| hsa-miR-4635 | MIMAT0019692 | 14.2 | 14.0 | 0.99 | -0.02 |
| hsa-miR-4284 | MIMAT0016915 | 15.6 | 14.0 | 0.90 | -0.16 |
| hsa-miR-532-5p | MIMAT0002888 |  | 14.0 |  |  |
| hsa-miR-3689b*, hsa-miR-3689c | MIMAT0018181, MIMAT0019007 | 25.0 | 14.0 | 0.56 | -0.84 |
| hsa-miR-219-2-3p | MIMAT0004675 |  | 14.0 |  |  |
| hsa-miR-3124-5p | MIMAT0014986 | 10.5 | 14.0 | 1.33 | 0.41 |
| hsa-miR-96* | MIMAT0004510 |  | 13.9 |  |  |
| hsa-miR-146b-5p | MIMAT0002809 | 20.9 | 13.9 | 0.67 | -0.59 |
| hsa-miR-320d | MIMAT0006764 | 16.9 | 13.9 | 0.82 | -0.28 |
| hsa-miR-4793-5p | MIMAT0019965 | 16.4 | 13.9 | 0.85 | -0.24 |
| hsa-miR-625 | MIMAT0003294 |  | 13.9 |  |  |
| hsa-miR-4724-5p | MIMAT0019841 | 8.6 | 13.9 | 1.62 | 0.70 |
| hsa-miR-148b | MIMAT0000759 |  | 13.9 |  |  |
| hsa-miR-509-3-5p | MIMAT0004975 |  | 13.8 |  |  |
| hsa-miR-3611 | MIMAT0017988 |  | 13.7 |  |  |
| hsa-miR-193a-5p | MIMAT0004614 | 10.4 | 13.7 | 1.31 | 0.39 |
| hsa-miR-3605-5p | MIMAT0017981 |  | 13.6 |  |  |
| hsa-miR-1271 | MIMAT0005796 | 30.6 | 13.6 | 0.44 | -1.17 |
| hsa-miR-4253 | MIMAT0016882 | 22.7 | 13.6 | 0.60 | -0.75 |
| hsa-miR-516a-5p | MIMAT0004770 |  | 13.6 |  |  |
| hsa-miR-324-5p | MIMAT0000761 |  | 13.5 |  |  |
| hsa-miR-4475 | MIMAT0019002 |  | 13.5 |  |  |
| hsa-miR-2355-3p | MIMAT0017950 |  | 13.5 |  |  |
| hsa-miR-3929 | MIMAT0018206 |  | 13.5 |  |  |
| hsa-miR-378* | MIMAT0000731 | 11.4 | 13.5 | 1.18 | 0.24 |
| hsa-miR-23a* | MIMAT0004496 | 16.8 | 13.5 | 0.80 | -0.32 |
| hsa-miR-362-3p | MIMAT0004683 |  | 13.4 |  |  |
| hsa-miR-132 | MIMAT0000426 |  | 13.4 |  |  |
| hsa-miR-670 | MIMAT0010357 |  | 13.4 |  |  |
| hsa-miR-3689a-3p | MIMAT0018118 |  | 13.4 |  |  |
| hsa-miR-1250 | MIMAT0005902 | 9.0 | 13.4 | 1.48 | 0.57 |
| hsa-miR-1295 | MIMAT0005885 |  | 13.3 |  |  |
| hsa-miR-4676-3p | MIMAT0019759 | 14.9 | 13.3 | 0.90 | -0.16 |
| hsa-miR-182 | MIMAT0000259 |  | 13.3 |  |  |
| hsa-miR-30c-2* | MIMAT0004550 | 9.8 | 13.3 | 1.37 | 0.45 |
| hsa-miR-4283 | MIMAT0016914 | 10.2 | 13.3 | 1.30 | 0.38 |
| hsa-miR-4295 | MIMAT0016844 | 12.6 | 13.3 | 1.05 | 0.07 |
| hsa-miR-422a | MIMAT0001339 |  | 13.3 |  |  |
| hsa-miR-609 | MIMAT0003277 |  | 13.3 |  |  |
| hsa-miR-485-5p | MIMAT0002175 |  | 13.2 |  |  |
| hsa-miR-3913-3p | MIMAT0019225 | 16.9 | 13.1 | 0.78 | -0.36 |
| hsa-miR-3150a-3p | MIMAT0015023 | 16.0 | 13.1 | 0.82 | -0.28 |
| hsa-miR-610 | MIMAT0003278 |  | 13.1 |  |  |
| hsa-miR-431* | MIMAT0004757 |  | 13.1 |  |  |
| hsa-miR-3680 | MIMAT0018106 |  | 13.1 |  |  |
| hsa-miR-1276 | MIMAT0005930 |  | 13.1 |  |  |
| hsa-miR-4316 | MIMAT0016867 |  | 13.0 |  |  |
| hsa-miR-598 | MIMAT0003266 | 9.1 | 13.0 | 1.43 | 0.52 |
| hsa-miR-3680* | MIMAT0018107 |  | 13.0 |  |  |
| hsa-miR-4450 | MIMAT0018971 | 20.0 | 13.0 | 0.65 | -0.62 |
| hsa-miR-4784 | MIMAT0019948 |  | 13.0 |  |  |
| hsa-miR-142-3p | MIMAT0000434 |  | 12.9 |  |  |
| hsa-miR-7-2* | MIMAT0004554 |  | 12.9 |  |  |
| hsa-miR-4252 | MIMAT0016886 | 14.7 | 12.9 | 0.88 | -0.19 |
| hsa-miR-4423-5p | MIMAT0019232 | 18.9 | 12.9 | 0.68 | -0.56 |
| hsa-miR-302c* | MIMAT0000716 | 11.8 | 12.9 | 1.09 | 0.12 |
| hsa-miR-4694-5p | MIMAT0019786 |  | 12.9 |  |  |
| hsa-miR-433 | MIMAT0001627 |  | 12.8 |  |  |
| hsa-miR-2117 | MIMAT0011162 | 13.3 | 12.8 | 0.96 | -0.06 |
| hsa-miR-921 | MIMAT0004971 | 9.2 | 12.8 | 1.38 | 0.47 |
| hsa-miR-3149 | MIMAT0015022 | 26.3 | 12.8 | 0.49 | -1.04 |
| hsa-miR-2277-5p | MIMAT0017352 |  | 12.8 |  |  |
| hsa-miR-3170 | MIMAT0015045 |  | 12.8 |  |  |
| hsa-miR-183 | MIMAT0000261 | 11.4 | 12.8 | 1.12 | 0.16 |
| hsa-miR-3138 | MIMAT0015006 | 13.1 | 12.7 | 0.97 | -0.04 |
| hsa-miR-449c* | MIMAT0013771 | 17.5 | 12.7 | 0.73 | -0.46 |
| hsa-miR-4280 | MIMAT0016911 |  | 12.7 |  |  |
| hsa-miR-33a | MIMAT0000091 |  | 12.7 |  |  |
| hsa-miR-4278 | MIMAT0016910 | 13.8 | 12.7 | 0.92 | -0.13 |
| hsa-miR-4422 | MIMAT0018935 | 13.5 | 12.6 | 0.94 | -0.09 |
| hsa-miR-122* | MIMAT0004590 |  | 12.6 |  |  |
| hsa-miR-676 | MIMAT0018204 | 9.4 | 12.6 | 1.34 | 0.42 |
| hsa-miR-4308 | MIMAT0016861 |  | 12.5 |  |  |
| hsa-miR-653 | MIMAT0003328 |  | 12.5 |  |  |
| hsa-miR-514b-5p | MIMAT0015087 | 14.6 | 12.5 | 0.86 | -0.22 |
| hsa-miR-4713-3p | MIMAT0019821 | 12.8 | 12.5 | 0.97 | -0.04 |
| hsa-miR-3649 | MIMAT0018069 |  | 12.4 |  |  |
| hsa-miR-429 | MIMAT0001536 |  | 12.4 |  |  |
| hsa-miR-127-3p | MIMAT0000446 | 11.6 | 12.4 | 1.06 | 0.09 |
| hsa-miR-339-3p | MIMAT0004702 |  | 12.3 |  |  |
| hsa-miR-4633-3p | MIMAT0019690 | 8.9 | 12.3 | 1.38 | 0.47 |
| hsa-miR-3927 | MIMAT0018202 |  | 12.3 |  |  |
| hsa-miR-138-2* | MIMAT0004596 |  | 12.2 |  |  |
| hsa-miR-147 | MIMAT0000251 |  | 12.2 |  |  |
| hsa-miR-4720-3p | MIMAT0019834 |  | 12.2 |  |  |
| hsa-miR-4529-3p | MIMAT0019068 | 18.4 | 12.2 | 0.66 | -0.59 |
| hsa-miR-365* | MIMAT0009199 | 17.1 | 12.2 | 0.71 | -0.50 |
| hsa-miR-1273c | MIMAT0015017 | 22.6 | 12.2 | 0.54 | -0.89 |
| hsa-miR-758 | MIMAT0003879 |  | 12.2 |  |  |
| hsa-miR-34c-3p | MIMAT0004677 | 19.4 | 12.1 | 0.62 | -0.68 |
| hsa-miR-375 | MIMAT0000728 |  | 12.0 |  |  |
| hsa-miR-676* | MIMAT0018203 | 9.5 | 12.0 | 1.26 | 0.33 |
| hsa-miR-513c | MIMAT0005789 | 12.6 | 11.9 | 0.95 | -0.07 |
| hsa-miR-4307 | MIMAT0016860 | 14.4 | 11.9 | 0.83 | -0.28 |
| hsa-miR-31 | MIMAT0000089 | 14.8 | 11.9 | 0.80 | -0.31 |
| hsa-miR-4517 | MIMAT0019054 |  | 11.9 |  |  |
| hsa-miR-4783-5p | MIMAT0019946 |  | 11.8 |  |  |
| hsa-miR-616* | MIMAT0003284 |  | 11.8 |  |  |
| hsa-miR-449b | MIMAT0003327 |  | 11.8 |  |  |
| hsa-miR-185 | MIMAT0000455 | 8.5 | 11.8 | 1.38 | 0.47 |
| hsa-miR-558 | MIMAT0003222 | 10.9 | 11.8 | 1.08 | 0.11 |
| hsa-miR-3157-5p | MIMAT0015031 |  | 11.8 |  |  |
| hsa-miR-4288 | MIMAT0016918 | 24.4 | 11.8 | 0.48 | -1.05 |
| hsa-miR-138-1* | MIMAT0004607 | 18.9 | 11.7 | 0.62 | -0.68 |
| hsa-miR-3152-5p | MIMAT0019207 |  | 11.7 |  |  |
| hsa-miR-4762-3p | MIMAT0019911 |  | 11.7 |  |  |
| hsa-miR-4650-3p | MIMAT0019714 |  | 11.7 |  |  |
| hsa-miR-378d | MIMAT0018926 | 11.3 | 11.7 | 1.03 | 0.04 |
| hsa-miR-371-3p | MIMAT0000723 |  | 11.6 |  |  |
| hsa-miR-548v | MIMAT0015020 |  | 11.6 |  |  |
| hsa-miR-193a-3p | MIMAT0000459 |  | 11.6 |  |  |
| hsa-miR-143* | MIMAT0004599 |  | 11.6 |  |  |
| hsa-miR-337-5p | MIMAT0004695 |  | 11.6 |  |  |
| hsa-miR-425 | MIMAT0003393 | 18.9 | 11.6 | 0.61 | -0.71 |
| hsa-miR-4794 | MIMAT0019967 | 11.6 | 11.5 | 1.00 | -0.01 |
| hsa-miR-451b | MIMAT0019840 |  | 11.5 |  |  |
| hsa-miR-548f | MIMAT0005895 |  | 11.5 |  |  |
| hsa-miR-3186-3p | MIMAT0015068 | 14.0 | 11.4 | 0.82 | -0.29 |
| hsa-miR-198 | MIMAT0000228 |  | 11.4 |  |  |
| hsa-miR-4264 | MIMAT0016899 |  | 11.4 |  |  |
| hsa-miR-4733-5p | MIMAT0019857 | 17.9 | 11.4 | 0.64 | -0.65 |
| hsa-miR-518c | MIMAT0002848 |  | 11.4 |  |  |
| hsa-miR-4451 | MIMAT0018973 |  | 11.3 |  |  |
| hsa-miR-4514 | MIMAT0019051 | 25.1 | 11.3 | 0.45 | -1.15 |
| hsa-miR-374a* | MIMAT0004688 |  | 11.3 |  |  |
| hsa-miR-4692 | MIMAT0019783 | 16.5 | 11.2 | 0.68 | -0.56 |
| hsa-miR-548c-5p | MIMAT0004806 |  | 11.2 |  |  |
| hsa-miR-3129-3p | MIMAT0019202 |  | 11.2 |  |  |
| hsa-miR-17* | MIMAT0000071 |  | 11.2 |  |  |
| hsa-miR-515-3p | MIMAT0002827 | 10.1 | 11.2 | 1.11 | 0.15 |
| hsa-miR-379* | MIMAT0004690 |  | 11.1 |  |  |
| hsa-miR-4660 | MIMAT0019728 | 12.9 | 11.1 | 0.86 | -0.21 |
| hsa-miR-153 | MIMAT0000439 |  | 11.1 |  |  |
| hsa-miR-33b* | MIMAT0004811 | 12.3 | 11.1 | 0.90 | -0.15 |
| hsa-miR-520d-5p | MIMAT0002855 |  | 11.1 |  |  |
| hsa-miR-216a | MIMAT0000273 |  | 11.1 |  |  |
| hsa-miR-3171 | MIMAT0015046 |  | 11.0 |  |  |
| hsa-miR-3921 | MIMAT0018196 |  | 11.0 |  |  |
| hsa-miR-4301 | MIMAT0016850 |  | 11.0 |  |  |
| hsa-miR-622 | MIMAT0003291 |  | 11.0 |  |  |
| hsa-miR-548s | MIMAT0014987 |  | 11.0 |  |  |
| hsa-miR-145 | MIMAT0000437 |  | 11.0 |  |  |
| hsa-miR-4633-5p | MIMAT0019689 | 23.6 | 11.0 | 0.46 | -1.11 |
| hsa-miR-15a | MIMAT0000068 |  | 11.0 |  |  |
| hsa-miR-4479 | MIMAT0019011 |  | 10.9 |  |  |
| hsa-miR-3150b-5p | MIMAT0019226 | 32.7 | 10.9 | 0.33 | -1.58 |
| hsa-miR-3664-5p | MIMAT0018086 |  | 10.9 |  |  |
| hsa-miR-4788 | MIMAT0019958 | 20.7 | 10.9 | 0.53 | -0.92 |
| hsa-miR-2682 | MIMAT0013517 |  | 10.9 |  |  |
| hsa-miR-378b | MIMAT0014999 | 12.8 | 10.9 | 0.85 | -0.24 |
| hsa-miR-1301 | MIMAT0005797 |  | 10.9 |  |  |
| hsa-miR-4255 | MIMAT0016885 |  | 10.9 |  |  |
| hsa-miR-130a* | MIMAT0004593 | 12.0 | 10.8 | 0.91 | -0.14 |
| hsa-miR-4797-3p | MIMAT0019973 |  | 10.8 |  |  |
| hsa-miR-30e* | MIMAT0000693 | 9.9 | 10.8 | 1.10 | 0.13 |
| hsa-miR-518d-3p | MIMAT0002864 |  | 10.8 |  |  |
| hsa-miR-568 | MIMAT0003232 |  | 10.8 |  |  |
| hsa-miR-183* | MIMAT0004560 |  | 10.8 |  |  |
| hsa-miR-4435 | MIMAT0018951 | 23.6 | 10.7 | 0.45 | -1.14 |
| hsa-miR-96 | MIMAT0000095 |  | 10.7 |  |  |
| hsa-miR-3692 | MIMAT0018122 |  | 10.7 |  |  |
| hsa-miR-3658 | MIMAT0018078 |  | 10.6 |  |  |
| hsa-miR-488 | MIMAT0004763 |  | 10.6 |  |  |
| hsa-miR-541* | MIMAT0004919 | 12.4 | 10.6 | 0.85 | -0.23 |
| hsa-miR-508-5p | MIMAT0004778 | 19.0 | 10.6 | 0.56 | -0.84 |
| hsa-miR-1269 | MIMAT0005923 |  | 10.6 |  |  |
| hsa-miR-129* | MIMAT0004548 | 12.2 | 10.6 | 0.87 | -0.21 |
| hsa-miR-4777-5p | MIMAT0019934 |  | 10.6 |  |  |
| hsa-miR-4425 | MIMAT0018940 | 11.6 | 10.5 | 0.90 | -0.15 |
| hsa-miR-502-3p | MIMAT0004775 |  | 10.5 |  |  |
| hsa-miR-20b* | MIMAT0004752 |  | 10.4 |  |  |
| hsa-miR-4778-3p | MIMAT0019937 |  | 10.4 |  |  |
| hsa-miR-544 | MIMAT0003164 |  | 10.4 |  |  |
| hsa-miR-1323 | MIMAT0005795 |  | 10.4 |  |  |
| hsa-miR-28-5p | MIMAT0000085 |  | 10.4 |  |  |
| hsa-miR-4802-3p | MIMAT0019982 |  | 10.4 |  |  |
| hsa-miR-891a | MIMAT0004902 | 15.6 | 10.4 | 0.66 | -0.59 |
| hsa-miR-329 | MIMAT0001629 |  | 10.4 |  |  |
| hsa-miR-4670-3p | MIMAT0019751 |  | 10.4 |  |  |
| hsa-miR-200c* | MIMAT0004657 | 9.6 | 10.4 | 1.08 | 0.11 |
| hsa-miR-1911* | MIMAT0007886 | 20.9 | 10.4 | 0.50 | -1.01 |
| hsa-miR-644 | MIMAT0003314 |  | 10.3 |  |  |
| hsa-miR-505 | MIMAT0002876 |  | 10.3 |  |  |
| hsa-miR-4499 | MIMAT0019035 | 10.0 | 10.3 | 1.03 | 0.04 |
| hsa-miR-221* | MIMAT0004568 |  | 10.3 |  |  |
| hsa-miR-141 | MIMAT0000432 |  | 10.3 |  |  |
| hsa-miR-1294 | MIMAT0005884 |  | 10.2 |  |  |
| hsa-miR-3691-5p | MIMAT0018120 | 8.5 | 10.2 | 1.20 | 0.26 |
| hsa-miR-4652-3p | MIMAT0019717 | 19.1 | 10.1 | 0.53 | -0.92 |
| hsa-miR-4452 | MIMAT0018974 |  | 10.1 |  |  |
| hsa-miR-3647-5p | MIMAT0018066 |  | 10.1 |  |  |
| hsa-miR-194 | MIMAT0000460 |  | 10.1 |  |  |
| hsa-miR-761 | MIMAT0010364 |  | 10.0 |  |  |
| hsa-miR-617 | MIMAT0003286 |  | 10.0 |  |  |
| hsa-miR-3065-5p | MIMAT0015066 |  | 10.0 |  |  |
| hsa-miR-1468 | MIMAT0006789 | 30.7 |  |  |  |
| hsa-miR-196b* | MIMAT0009201 | 27.9 |  |  |  |
| hsa-miR-518b | MIMAT0002844 | 26.4 |  |  |  |
| hsa-miR-151b | MIMAT0010214 | 25.6 |  |  |  |
| hsa-let-7g | MIMAT0000414 | 24.3 |  |  |  |
| hsa-miR-1292 | MIMAT0005943 | 24.2 |  |  |  |
| hsa-miR-19a | MIMAT0000073 | 24.0 |  |  |  |
| hsa-miR-130b* | MIMAT0004680 | 23.5 |  |  |  |
| hsa-miR-3922-3p | MIMAT0018197 | 22.1 |  |  |  |
| hsa-miR-3681* | MIMAT0018109 | 21.4 |  |  |  |
| hsa-miR-3685 | MIMAT0018113 | 21.2 |  |  |  |
| hsa-miR-1285 | MIMAT0005876 | 20.4 |  |  |  |
| hsa-miR-3609 | MIMAT0017986 | 20.1 |  |  |  |
| hsa-miR-105 | MIMAT0000102 | 19.5 |  |  |  |
| hsa-miR-3137 | MIMAT0015005 | 19.5 |  |  |  |
| hsa-miR-3654 | MIMAT0018074 | 19.0 |  |  |  |
| hsa-miR-3660 | MIMAT0018081 | 18.4 |  |  |  |
| hsa-miR-769-5p | MIMAT0003886 | 18.0 |  |  |  |
| hsa-miR-4781-5p | MIMAT0019942 | 17.7 |  |  |  |
| hsa-miR-660 | MIMAT0003338 | 17.5 |  |  |  |
| hsa-miR-150 | MIMAT0000451 | 17.4 |  |  |  |
| hsa-miR-3177-3p | MIMAT0015054 | 17.4 |  |  |  |
| hsa-miR-2681* | MIMAT0013515 | 17.3 |  |  |  |
| hsa-miR-647 | MIMAT0003317 | 17.0 |  |  |  |
| hsa-miR-4768-3p | MIMAT0019921 | 16.9 |  |  |  |
| hsa-miR-1182 | MIMAT0005827 | 16.8 |  |  |  |
| hsa-miR-222* | MIMAT0004569 | 16.6 |  |  |  |
| hsa-miR-3936 | MIMAT0018351 | 16.5 |  |  |  |
| hsa-miR-3064-3p | MIMAT0019865 | 16.5 |  |  |  |
| hsa-miR-29b | MIMAT0000100 | 16.5 |  |  |  |
| hsa-miR-4785 | MIMAT0019949 | 16.4 |  |  |  |
| hsa-miR-27b | MIMAT0000419 | 16.4 |  |  |  |
| hsa-miR-412 | MIMAT0002170 | 16.1 |  |  |  |
| hsa-miR-541 | MIMAT0004920 | 16.0 |  |  |  |
| hsa-miR-4773 | MIMAT0019928 | 16.0 |  |  |  |
| hsa-miR-106b* | MIMAT0004672 | 16.0 |  |  |  |
| hsa-miR-3945 | MIMAT0018361 | 15.9 |  |  |  |
| hsa-miR-3924 | MIMAT0018199 | 15.9 |  |  |  |
| hsa-miR-605 | MIMAT0003273 | 15.7 |  |  |  |
| hsa-miR-548i | MIMAT0005935 | 15.2 |  |  |  |
| hsa-miR-938 | MIMAT0004981 | 14.9 |  |  |  |
| hsa-miR-4716-3p | MIMAT0019827 | 14.8 |  |  |  |
| hsa-miR-500b | MIMAT0016925 | 14.8 |  |  |  |
| hsa-miR-24-2* | MIMAT0004497 | 14.7 |  |  |  |
| hsa-miR-548d-5p | MIMAT0004812 | 14.6 |  |  |  |
| hsa-miR-337-3p | MIMAT0000754 | 14.6 |  |  |  |
| hsa-miR-208a | MIMAT0000241 | 14.4 |  |  |  |
| hsa-miR-4256 | MIMAT0016877 | 14.4 |  |  |  |
| hsa-miR-770-5p | MIMAT0003948 | 14.3 |  |  |  |
| hsa-miR-1184 | MIMAT0005829 | 14.2 |  |  |  |
| hsa-miR-3074-3p | MIMAT0015027 | 14.1 |  |  |  |
| hsa-miR-4681 | MIMAT0019766 | 14.0 |  |  |  |
| hsa-miR-4733-3p | MIMAT0019858 | 13.9 |  |  |  |
| hsa-miR-151-3p | MIMAT0000757 | 13.8 |  |  |  |
| hsa-miR-1270 | MIMAT0005924 | 13.8 |  |  |  |
| hsa-miR-509-3p | MIMAT0002881 | 13.8 |  |  |  |
| hsa-miR-601 | MIMAT0003269 | 13.8 |  |  |  |
| hsa-miR-1208 | MIMAT0005873 | 13.8 |  |  |  |
| hsa-miR-662 | MIMAT0003325 | 13.7 |  |  |  |
| hsa-miR-3132 | MIMAT0014997 | 13.6 |  |  |  |
| hsa-miR-4431 | MIMAT0018947 | 13.5 |  |  |  |
| hsa-miR-125b-2* | MIMAT0004603 | 13.4 |  |  |  |
| hsa-miR-635 | MIMAT0003305 | 13.3 |  |  |  |
| hsa-miR-4526 | MIMAT0019065 | 13.3 |  |  |  |
| hsa-miR-2964a-5p | MIMAT0019747 | 13.3 |  |  |  |
| hsa-miR-3127-3p | MIMAT0019201 | 13.2 |  |  |  |
| hsa-miR-4300 | MIMAT0016853 | 13.2 |  |  |  |
| hsa-miR-519e | MIMAT0002829 | 13.1 |  |  |  |
| hsa-miR-5095 | MIMAT0020600 | 13.1 |  |  |  |
| hsa-miR-148a | MIMAT0000243 | 12.9 |  |  |  |
| hsa-miR-26b | MIMAT0000083 | 12.9 |  |  |  |
| hsa-miR-1287 | MIMAT0005878 | 12.8 |  |  |  |
| hsa-miR-3941 | MIMAT0018357 | 12.8 |  |  |  |
| hsa-miR-1178 | MIMAT0005823 | 12.7 |  |  |  |
| hsa-miR-1 | MIMAT0000416 | 12.7 |  |  |  |
| hsa-miR-767-5p | MIMAT0003882 | 12.6 |  |  |  |
| hsa-miR-7 | MIMAT0000252 | 12.5 |  |  |  |
| hsa-miR-4531 | MIMAT0019070 | 12.4 |  |  |  |
| hsa-miR-411* | MIMAT0004813 | 12.4 |  |  |  |
| hsa-miR-802 | MIMAT0004185 | 12.4 |  |  |  |
| hsa-miR-664* | MIMAT0005948 | 12.3 |  |  |  |
| hsa-miR-888 | MIMAT0004916 | 12.3 |  |  |  |
| hsa-miR-539 | MIMAT0003163 | 12.1 |  |  |  |
| hsa-miR-4757-3p | MIMAT0019902 | 12.1 |  |  |  |
| hsa-miR-589* | MIMAT0003256 | 12.1 |  |  |  |
| hsa-miR-3074-5p | MIMAT0019208 | 12.0 |  |  |  |
| hsa-miR-3688-3p | MIMAT0018116 | 11.9 |  |  |  |
| hsa-miR-4659b-3p | MIMAT0019734 | 11.9 |  |  |  |
| hsa-miR-4285 | MIMAT0016913 | 11.9 |  |  |  |
| hsa-miR-378h | MIMAT0018984 | 11.8 |  |  |  |
| hsa-miR-892a | MIMAT0004907 | 11.8 |  |  |  |
| hsa-miR-4769-5p | MIMAT0019922 | 11.8 |  |  |  |
| hsa-miR-367 | MIMAT0000719 | 11.7 |  |  |  |
| hsa-miR-4426 | MIMAT0018941 | 11.7 |  |  |  |
| hsa-miR-4753-5p | MIMAT0019890 | 11.5 |  |  |  |
| hsa-miR-552 | MIMAT0003215 | 11.4 |  |  |  |
| hsa-miR-203 | MIMAT0000264 | 11.3 |  |  |  |
| hsa-miR-518c* | MIMAT0002847 | 11.2 |  |  |  |
| hsa-miR-4709-5p | MIMAT0019811 | 11.1 |  |  |  |
| hsa-miR-4709-3p | MIMAT0019812 | 11.1 |  |  |  |
| hsa-miR-432 | MIMAT0002814 | 11.1 |  |  |  |
| hsa-miR-302e | MIMAT0005931 | 11.0 |  |  |  |
| hsa-miR-4677-3p | MIMAT0019761 | 10.9 |  |  |  |
| hsa-miR-589 | MIMAT0004799 | 10.9 |  |  |  |
| hsa-miR-497 | MIMAT0002820 | 10.7 |  |  |  |
| hsa-miR-4468 | MIMAT0018995 | 10.7 |  |  |  |
| hsa-miR-1205 | MIMAT0005869 | 10.7 |  |  |  |
| hsa-miR-3166 | MIMAT0015040 | 10.7 |  |  |  |
| hsa-miR-3529 | MIMAT0019828 | 10.7 |  |  |  |
| hsa-miR-1226* | MIMAT0005576 | 10.6 |  |  |  |
| hsa-miR-4737 | MIMAT0019863 | 10.6 |  |  |  |
| hsa-miR-218 | MIMAT0000275 | 10.5 |  |  |  |
| hsa-miR-4477b | MIMAT0019005 | 10.5 |  |  |  |
| hsa-miR-4325 | MIMAT0016887 | 10.5 |  |  |  |
| hsa-miR-544b | MIMAT0015004 | 10.5 |  |  |  |
| hsa-miR-4755-5p | MIMAT0019895 | 10.4 |  |  |  |
| hsa-miR-340* | MIMAT0000750 | 10.4 |  |  |  |
| hsa-miR-1911 | MIMAT0007885 | 10.4 |  |  |  |
| hsa-miR-4418 | MIMAT0018930 | 10.4 |  |  |  |
| hsa-miR-517a | MIMAT0002852 | 10.4 |  |  |  |
| hsa-miR-708* | MIMAT0004927 | 10.4 |  |  |  |
| hsa-miR-4691-3p | MIMAT0019782 | 10.3 |  |  |  |
| hsa-miR-1273g | MIMAT0020602 | 10.3 |  |  |  |
| hsa-miR-4460 | MIMAT0018982 | 10.2 |  |  |  |
| hsa-miR-556-3p | MIMAT0004793 | 10.2 |  |  |  |
| hsa-miR-181a* | MIMAT0000270 | 10.2 |  |  |  |
| hsa-miR-3667-5p | MIMAT0018089 | 10.1 |  |  |  |
| hsa-miR-3675-5p | MIMAT0018098 | 10.1 |  |  |  |
| hsa-miR-3926 | MIMAT0018201 | 10.1 |  |  |  |
| hsa-miR-4718 | MIMAT0019831 | 10.1 |  |  |  |
| hsa-miR-181d | MIMAT0002821 | 10.0 |  |  |  |
| hsa-miR-297 | MIMAT0004450 | 10.0 |  |  |  |
| hsa-miR-942 | MIMAT0004985 | 10.0 |  |  |  |
| hsa-miR-1183 | MIMAT0005828 | 9.9 |  |  |  |
| hsa-miR-491-3p | MIMAT0004765 | 9.9 |  |  |  |
| hsa-miR-455-3p | MIMAT0004784 | 9.9 |  |  |  |
| hsa-miR-4524* | MIMAT0019063 | 9.9 |  |  |  |
| hsa-miR-3153 | MIMAT0015026 | 9.7 |  |  |  |
| hsa-miR-16-2* | MIMAT0004518 | 9.7 |  |  |  |
| hsa-miR-604 | MIMAT0003272 | 9.7 |  |  |  |
| hsa-miR-4439 | MIMAT0018957 | 9.7 |  |  |  |
| hsa-miR-4490 | MIMAT0019025 | 9.6 |  |  |  |
| hsa-miR-4493 | MIMAT0019028 | 9.6 |  |  |  |
| hsa-miR-4684-5p | MIMAT0019769 | 9.6 |  |  |  |
| hsa-let-7f-2* | MIMAT0004487 | 9.6 |  |  |  |
| hsa-miR-3907 | MIMAT0018179 | 9.6 |  |  |  |
| hsa-miR-4745-3p | MIMAT0019879 | 9.5 |  |  |  |
| hsa-miR-514b-3p | MIMAT0015088 | 9.5 |  |  |  |
| hsa-miR-4503 | MIMAT0019039 | 9.4 |  |  |  |
| hsa-miR-155 | MIMAT0000646 | 9.4 |  |  |  |
| hsa-miR-602 | MIMAT0003270 | 9.4 |  |  |  |
| hsa-miR-10b | MIMAT0000254 | 9.3 |  |  |  |
| hsa-miR-888* | MIMAT0004917 | 9.3 |  |  |  |
| hsa-miR-3681 | MIMAT0018108 | 9.3 |  |  |  |
| hsa-miR-4441 | MIMAT0018959 | 9.2 |  |  |  |
| hsa-miR-154* | MIMAT0000453 | 9.2 |  |  |  |
| hsa-miR-19b-2* | MIMAT0004492 | 9.0 |  |  |  |
| hsa-miR-373* | MIMAT0000725 | 8.9 |  |  |  |
| hsa-miR-519c-5p, hsa-miR-523*, hsa-miR-518e*, hsa-miR-522*, hsa-miR-519a*, hsa-miR-519b-5p | MIMAT0002831, MIMAT0005449, MIMAT0005450, MIMAT0005451, MIMAT0005452, MIMAT0005454 | 8.8 |  |  |  |
| hsa-miR-520g | MIMAT0002858 | 8.8 |  |  |  |
| hsa-miR-4799-5p | MIMAT0019976 | 8.8 |  |  |  |
| hsa-miR-4427 | MIMAT0018942 | 8.8 |  |  |  |
| hsa-miR-876-5p | MIMAT0004924 | 8.7 |  |  |  |
| hsa-miR-1273 | MIMAT0005926 | 8.7 |  |  |  |
| hsa-miR-3152-3p | MIMAT0015025 | 8.7 |  |  |  |
| hsa-miR-495 | MIMAT0002817 | 8.6 |  |  |  |
| hsa-miR-554 | MIMAT0003217 | 8.6 |  |  |  |
| hsa-miR-363 | MIMAT0000707 | 8.6 |  |  |  |
| hsa-miR-4764-3p | MIMAT0019915 | 8.6 |  |  |  |
| hsa-miR-520f | MIMAT0002830 | 8.6 |  |  |  |
| hsa-miR-4712-3p | MIMAT0019819 | 8.5 |  |  |  |
| hsa-miR-549 | MIMAT0003333 | 8.5 |  |  |  |
| hsa-miR-3201 | MIMAT0015086 | 8.5 |  |  |  |
